# Study on the drop impact characteristics and impact damage mechanism of sweet potato tubers during harvest

**DOI:** 10.1371/journal.pone.0255856

**Published:** 2021-08-24

**Authors:** Guocheng Bao, Gongpu Wang, Bing Wang, Lianglong Hu, Xiaowei Xu, Haiyang Shen, Longlong Ji

**Affiliations:** 1 Agricultural Products Harvest and Postpartum Processing Engineering Technology Research Center, Nanjing Institute of Agricultural Mechanization, Ministry of Agriculture and Rural Affairs, Nanjing, Jiangsu, China; 2 Hunan Agricultural Equipment Research Institute, Changsha, Hunan, China; Al Mansour University College-Baghdad, IRAQ

## Abstract

Collision of falling in the mechanical harvesting process of sweet potato is one of the main causes of epidermal destruction and damage to sweet potato tubers. Therefore, a sweet potato mechanical characteristic test and a full-factor sweet potato drop test were designed. Based on the analysis of the fitting mathematical model, the impact of the drop height, collision material and sweet potato chunk size on the damage of the sweet potato were studied. The mathematical models were established by fitting analysis of the IBM SPSS Statistics 22 software between the drop height and the sweet potato chunk size with each test index (impact force, impact stress, broken skin area and damaged area). The critical epidermal destruction height and the critical damage height of a certain size of sweet potato when it collides with a collision material can be calculated by the mathematical model, and the critical epidermal destruction mass and critical damage mass of sweet potato when it falls from a certain height and collides with a collision material can also be calculated. Then a series of critical values (including critical epidermal destruction force value, critical epidermal destruction impact stress, critical damage force value, critical damage impact stress) of mechanical properties of sweet potato were obtained. The results show that the impact deformation of sweet potato includes both elastic and plastic ones, and has similar stress relaxation characteristics. The critical damage impact stress of sweet potato is that the average value of the impact stress on the contact surface is less than it’s Firmness. The results provided a theoretical basis for understanding the collision damage mechanism of sweet potato and how to reduce the damage during harvest.

## Introduction

Sweet potato is an important food crop and energy crop, as well as a high-quality anti-cancer health food [1–3]. China’s sweet potato planting area and output rank first in the world. However, compared with developed countries, China’s sweet potato production mechanization process started late and invested less [4–10]. Although it has developed rapidly, there remains a significant gap. Mechanized production technology is relatively backward and the degree of high efficiency and serialization of work tools is still low [11–14], especially in the harvest link where the labor intensity is the highest, a large amount of labor is still used [14]. During mechanized harvesting, sweet potatoes inevitably collide and collide with the conveying and separating parts of the machine, soil blocks, and potato collection containers, which will cause mechanical damage to the potato chunks [15]. According to the "DB41/T1010-2015 Technical Regulations for Mechanized Ridging and Harvesting of Sweet Potatoes", the mechanical damage of potato chunks is divided into sweet potatoes with epidermal destruction and broken sweet potatoes [16]. Therefore, studying the impact characteristics and damage mechanism of potato chunks in the process of falling and dropping provides a reference for the design and optimization of sweet potato harvesting machinery, which will effectively reduce the damage to potato chunks in the mechanized sweet potato harvesting process, and is of great significance in promoting the mechanization of the sweet potato industry.

Mechanical damage of fruits and vegetables has attracted the attention of many scholars. At present, there are relatively many related studies on the drop characteristics of potatoes, apples, peaches and other fruits and vegetables, and there are few studies on the collision mechanics characteristics of sweet potatoes. According to Kleinhenz (2001), growers lose up to 20% of their income due to impact injuries during harvesting and subsequent handling operations. Mechanical damage reduces market price, promotes shrinkage in storage (due to a relatively high respiration rate of injured tissues linked with increased water loss), and increased processing and handling costs [17]. Gao GH and et al (2019) established a mathematical model between the drop height and impact force, drop height and impact stress through a mechanical properties test and drop impact test of sweet potato, and measured the damage force value and critical drop damage height of a certain quality range of sweet potato. However, the influencing factors he considered were relatively single, and no measures were taken to control the weight difference between potato tuber in the sample, which increased the test error [18]. Xie SS and et al (2020) analyzed the impact of test factors on the volume of potato collision damage through orthogonal experiments; combined with the potato collision acceleration change curve, analyzed the collision compression process between the potato and the rod; and selected the initial height and potato mass as the test factors to investigate the collision acceleration; The peak value changed with the level of various factors and a regression model is established. Finally, the critical value of potato collision damage was tested. The experiment used an acceleration sensor for data acquisition. During his experiment, a hole was dug in the center of the potato tuber and the sensor was placed inside the hole. Because the sensor was wired transmission, the connecting wire would be stressed to interfere with the experimental results. The meat inside the potato tuber was soft and the hole had gaps, so the measurement was uncertain [19]. Sang YY and et al (2008) conducted experimental research on potato collision damage to obtain the safe drop height of the potato, and simulated the collision and analyzed the collision stress value using finite elements. In their test, the damage interval of potato tuber was not clearly defined, and the potato tuber model for collision simulation was standard spherical, so the simulation collision was too idealized and there was a big gap between the effect of actual collision [20]; Nuri Caglayan and et al (2018), similar simulation research was done with Sang YY to analyzed the stress distribution during the drop collision [21]. R. Mathew and G. M. Hyde (1997) proposed the pendulum test platform that was used to convert the vertical impact of potato into horizontal impact, and the acceleration signal during collision was collected. However, no in-depth study on the change of physical quantity was conducted, and the selection of test factors was not well matched in the influencing factors of mechanized harvest of potato [22]. Hong X and et al (2012) proposed a method to determine the critical damage drop height of the potato. Methods: Through the static compression test of the whole potato, the compression deformation variable during damage was obtained, and the relationship model between different drop heights and deformation variables was obtained through the drop impact test, to obtain the critical damage drop height of the potato. During his test, the whole potato was loaded to detect the deformation on both sides of the potato tuber. However, the shape of potato tuber was not regular, and the amount of deformation on both sides was different. Moreover, the compression test and collision test used different samples, and the difference in contact area and characteristics between them were going to have an impact on the experimental analysis [23]. Ahmed Mustafa Rady and Soliman N. Soliman (2015) studied the collision damage law between steel sheet, steel rods, and coated steel rods [24].

This paper takes sweet potato as the research object, through the sweet potato mechanical properties test and drop impact test, the mathematical model between the drop height and impact force, impact stress, broken skin area, damaged area was established respectively. The mathematical models between the weight of sweet potato and impact force, impact stress, broken skin area and damaged area were established. The theoretical analysis is based on fitting mathematical models. The influencing factors were fully considered in the design of the test, and corresponding measures were taken to reduce the test error. The experimental system read the value of impact force in each millisecond of the sweet potato collision process, and based on this, analyzed the relationship between the movement process and the degree of damage, and searched for the damage mechanism and the characteristic value of physical parameters of the sweet potato. The research results provide a more scientific theoretical basis and data reference for the design and optimization of sweet potato harvesting, processing, storage, quality inspection and other related equipment [25–28].

## Materials and methods

### Test materials

For the experiment, the "Sweet Potato Su 16" grown in the experimental field of the Baima Experimental Base of the Nanjing Agricultural Mechanization Research Institute of the Ministry of Agriculture and Rural Affairs was selected as the research object, and the sweet potato with no pests, diseases and damage was selected as the test object. The moisture content of sweet potatoes was 76.44%. The test potatoes refer to the "People’s Republic of China Agricultural Industry Standard-Sweet Potato Grade Specifications" and the "Sweet Potato Su 16" potato quality distribution, taking 5 quality points of sweet potato as the research object [29]. According to the actual mechanical harvesting of sweet potato collision objects in the field, the test collision materials were selected: 65Mn steel, plastic baskets, rubber, sweet potatoes, and soil blocks. The material characteristics are shown in Table 1. The test soil block was taken from the test field of the Baima Test Base of The Nanjing Institute of Agricultural Mechanization, Ministry of Agriculture and Rural Affairs. The soil type is sandy loam soil with partial stickiness, which mainly includes massive and aggregate structures. Its characteristic parameters were: water content of 13.61%, firmness of 302 kPa, and cohesive force of 0.01 MPa. The height of the soil block sample was 100 mm and the diameter was 85 mm.

**Table 1:** Physical properties of collision materials.

Material type	Thickness/(mm)	Density/(g·cm^-3^)	Elastic Modulus/(MPa)	Poisson’s ratio
65Mn steel	12	7.81	2.1×10^5^	0.3
plastic basket	4	0.96	900	0.38
rubber plate	4	1.8	100	0.3
sweet potato	-	1.13	3.71	0.41
soil block	-	2.6	35	0.44

Material parameters can be measured by special testing instruments or obtained by referring to material parameter manual.

### Main equipment

**Fig 1:**
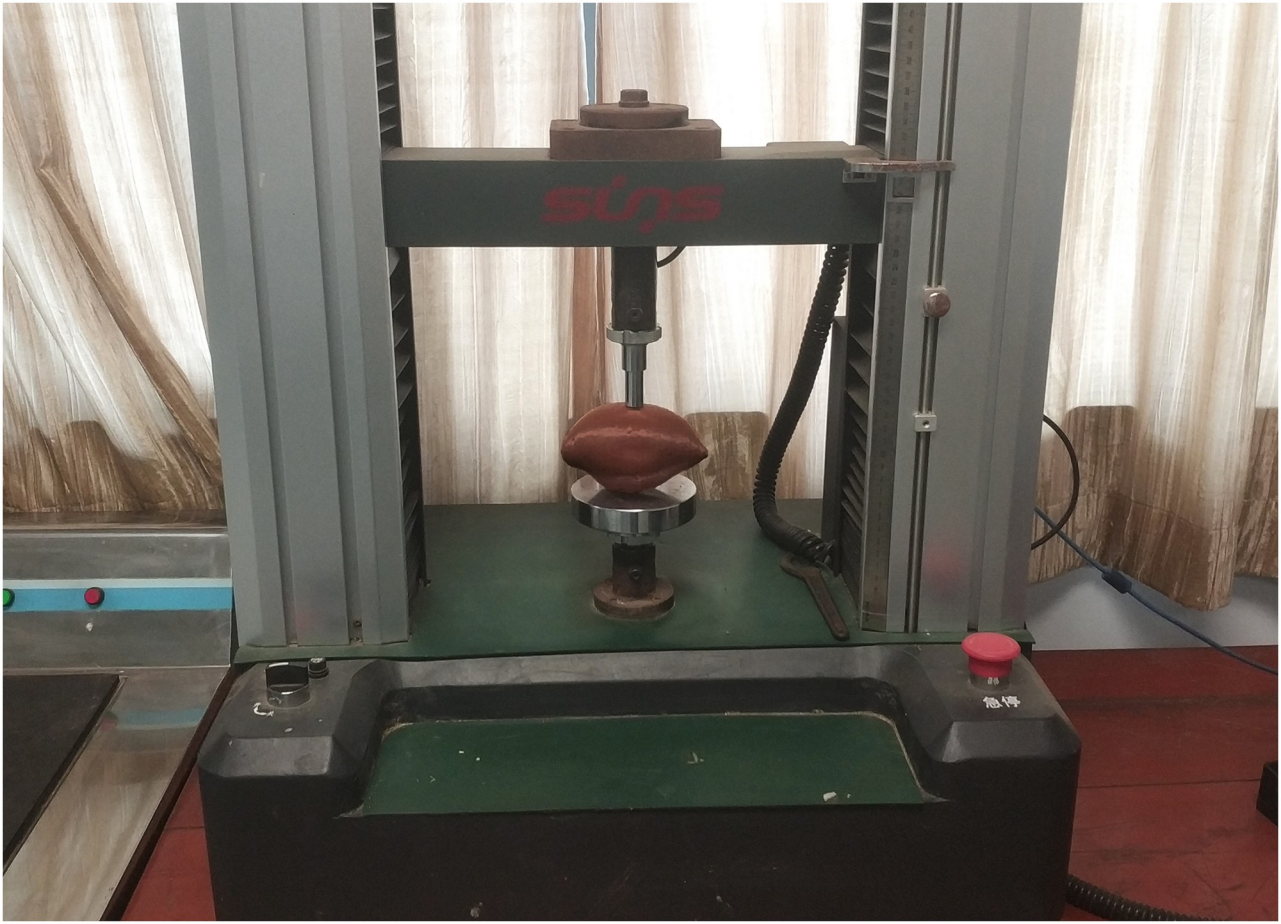
DWD type electronic universal testing machine.

The instrument used for sweet potato firmness test mainly included the Shenzhen Sansi DWD type electronic universal testing machine (Fig 1). Its rated load was 5KN, accuracy grade was 0.5, displacement resolution was 0.01mm, and loading rate was 0.01~500mm/min. The load-displacement relationship can be automatically recorded by the computer by tracing points, and the coordinates and structural parameters of each point can be read out from the designated file [30], electronic balance (range 500g, accuracy 0.01g), digital display vernier caliper (range 150mm, accuracy 0.01mm), Utility knife, etc.

**Fig 2:**
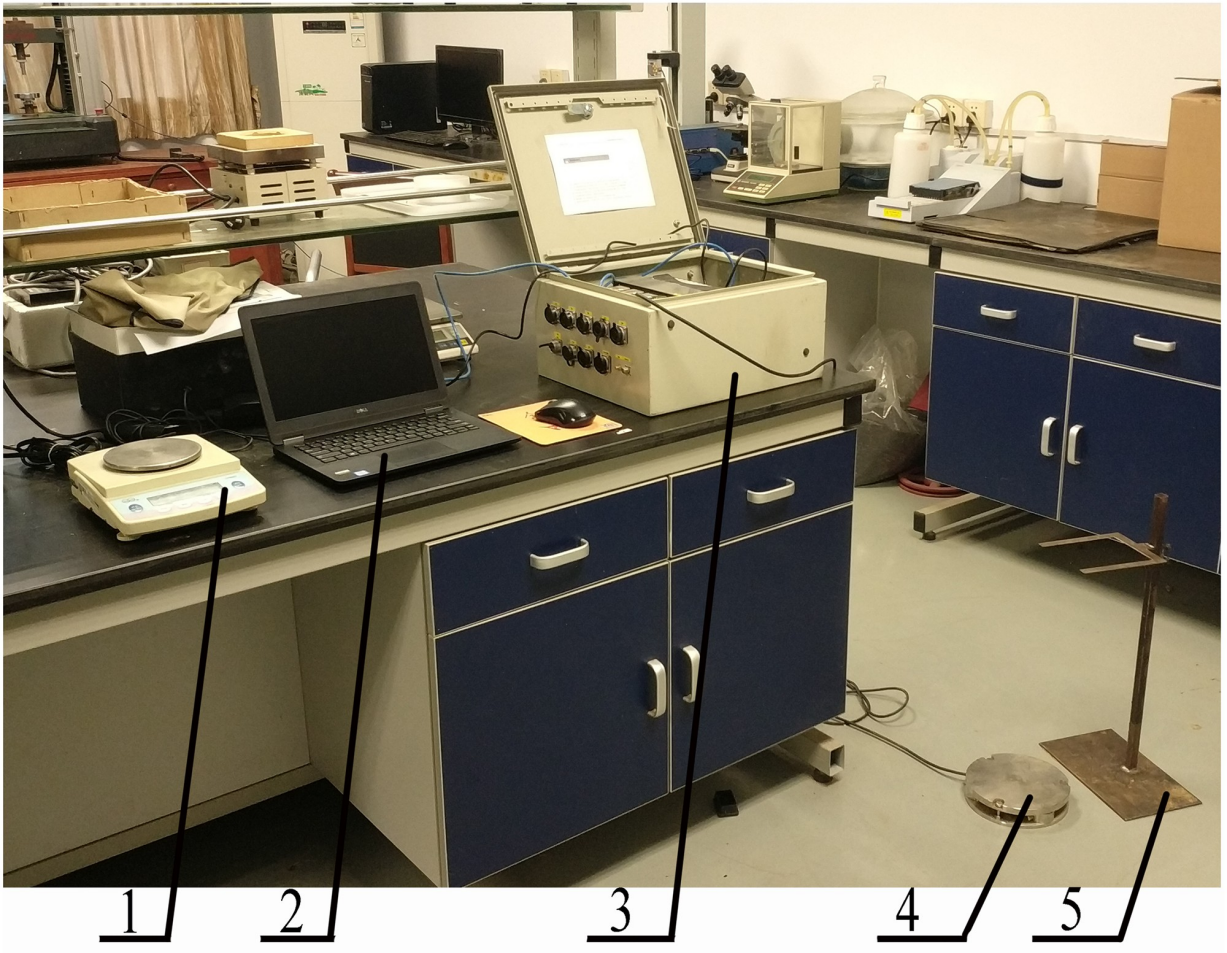
Sweet potato drop impact test system. 1. Electronic balance 2. Laptop 3. Signal amplifier 4. impact force sensor platform 5. Ruler frame.

The instruments used in the sweet potato drop impact test are mainly self-built sweet potato drop impact test system (Fig 2). In order to prevent the sweet potato falling impact from overturning due to the fact that landing point is not in the center of the impact plane, three M1011 impact sensors are placed and connected in a triangle with a combined measuring range of 2kN and a sampling frequency of 15kHz. Using MX840B high-speed data acquisition instrument, the single sampling rate of each channel is up to 40KS/s, low-level effective, 24-bit A/D converter. A laptop.

### Test method

Determination of sweet potato firmness: DWD electronic universal testing machine was used to load the whole sweet potato sample with a flat top cylindrical indenter. The loading speed was 30mm/min, and a 15mm diameter indenter was selected. First, vernier caliper was used to measure the characteristic size of sweet potato sample, and then the measured sweet potato sample was placed horizontally in the fixture of electronic universal testing machine along the long axis, The test device and loading mode are shown in Fig 1. According to the research on the mechanical and physical properties of sweet potato during harvest period by Shen Haiyang and others, the difference between the axial and radial mechanical properties of sweet potato is small, which can be regarded as an isotropic material [31]. Therefore, this experiment only studied the single axial firmness characteristics of sweet potato. In order to reduce the experimental error caused by the particularity of individual samples and the subjective factors of the experimentally, every sample was randomly selected from each level of sweet potato drop impact test. Five groups of pressure displacement curves were obtained, and the first mutation value was determined. The firmness of sweet potato was calculated using formula (1) and the average value was calculated.

$$\sigma_{\;pole}=\frac{F_{\;pole}}{\pi r^{2}}$$ (1)

where: *F*_*pole*_ is the maximum value of the pressure on the sample, N;

*r* is the radius of the cylindrical indenter, mm.

Drop impact test of sweet potato: a drop impact test system was established. The drop height h, collision material X and chunk size m of sweet potatoes were taken as test factors, and the drop impact characteristics (impact force peak F, acceleration peak A and maximum deformation s) and damage (the area of epidermal destruction Y1 and the area of broken Y2) of sweet potato were taken as test indexes for the full factor test (Table 2), Five sweet potato samples of similar quality were selected for each group of test, and the sweet potatoes were controlled near the tests quality level with art knife [32,33]. Before the test, each sample was weighed and measured, and the mass m was recorded and labeled. During the test, the sweet potato was placed at the specified drop height (Table 2), and the center of gravity of the sweet potato was above the triangle center of the sensor for free drop test. Each sweet potato sample was rotated 180° along the long axis and then tested again for a total of 10 tests. The area of epidermal destruction and the area of broken of each sweet potato sample were measured with transparent mesh paper, and the average area of each group was calculated as the test index. The impact force F-time T signal during each experiment were collected by the software test system, and a group of signals closest to the average impact force value were selected for analysis to study the influence level of various factors on the indicators [34]. After each drop impact rebound, the sweet potato sample was immediately taken to prevent the damage caused by the second drop impact.

**Table 2:** Levels of test factors.

factor	Level				
	1	2	3	4	5
Drop height/mm	100	200	300	400	500
Collision material	65Mn steel	plastic basket	rubber plate	sweet potato	soil block
Potato chunk size/g	100	200	300	400	500

The table lists five levels of parameter setting for the three factors in the test.

### Experimental principle

Through the sweet potato firmness test, the sweet potato firmness *σ*_*pole*_ was calculated by formula (1), and the average firmness is taken.

The sweet potato drop impact test system can measure the relationship between impact force *F* and time *t* when potato pieces of different quality fall at different heights and collide with different materials. Sweet potato m falls freely from height h and collides with the collision material *x* fixed on the sensor tray. The impact force *F*-time *t* signal during the impact was collected. If the influence of air resistance is neglected, the kinematic equation and related mathematical formulas can be used. The relationship among the impact force *F*, acceleration *a*, velocity *v*, and impact compression deformation *s* received by the sweet potato sample is [22,35]:

$$a=g-\frac{F}{m}$$ (2)

$$v_{0}=\sqrt{2gh}$$ (3)

$$v=v_{0}+\int_{0}^{t}adt$$ (4)

$$s_{0}=\int_{0}^{t}vdt$$ (5)

$$s=s_{0}-s_{1}$$ (6)

where:

*a* is the falling collision acceleration of the potato piece, m/s^2^;

*g* is the acceleration of gravity, taking g = 9.8 m/s^2^;

*h* is the falling height of the potato piece, mm;

*v*_*0*_ is the instantaneous velocity when the potato piece just touches the collision material, m/s;

*v* is the impact velocity of the potato chunks, m/s;

*s* is the impact compression deformation of the potato chunks, mm;

*s*_*0*_ is the displacement of the center of gravity of the potato chunks, mm;

*s*_*1*_ is the deformation of the collision material, mm.

**Fig 3:**
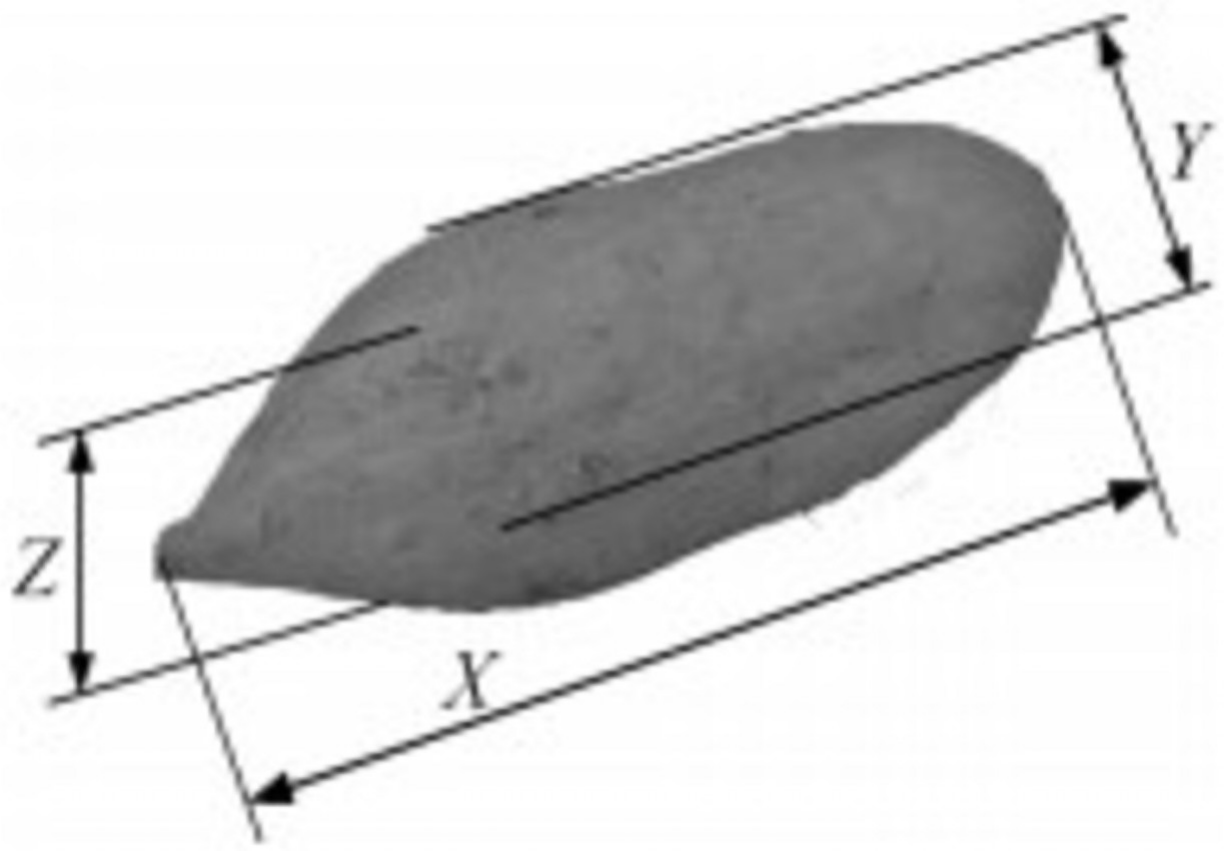
Characteristic dimensions of sweet potato. Calibrate the size characteristics of sweet potato.

The harvest time of sweet potato in China is mostly in October because of the variety of sweet potato, there are great differences among different varieties. The sweet potato variety "sweet potato Su 16" used in this experiment has a longer potato type, which is similar to long spindle. In order to facilitate the experimental operation and theoretical analysis, the ellipsoid is used as the model to analyze the impact shape of sweet potato. The characteristic dimensions are shown in Fig 3. The short axis 2*B* and the short axis 2*C* correspond to the average characteristic dimensions *y* and *Z* respectively. Because the two ends of the spindle are long, the long axis 2*A* should be slightly less than *x*, and *S* is the impact shape variable of sweet potato, according to the ellipsoid model equation, the relationship between the contact area *a* and the shape variable *s* is obtained, as shown in Eq 7.


$$A=\pi ab[1-\frac{{\left(c-s\right)}^{2}}{c^{2}}]$$ (7)

From the above model and formula 7, the impact stress of sweet potato can be calculated when it falls freely from a certain height, as shown in formula 8.


$$\sigma =\frac{F}{A}$$ (8)

## Results and discussion

In order to fit the actual motion and throwing motion of sweet potato on the harvester, a group of experiments of sweet potato falling along the long axis were added. However, by observing the impact position of sweet potato and impact material, it was found that whether the sweet potato fell along the long axis or short axis, even if the sweet potato fell along the same axis, the position of tuber was different when it collided with impact material. Because the shape of sweet potato is similar to a long spindle and the shape of sweet potato is irregular, the sweet potato will automatically adjust its posture due to the position of the center of gravity in the process of free fall, resulting in a certain rotation. When the sweet potato falls vertically, the two ends of most sweet potato first contact the impact surface and then act as a fulcrum, which makes the sweet potato deflect and finally injure the side of the sweet potato. Therefore, this study focuses on the analysis of sweet potato side impact characteristics [36–38].

**Fig 4:**
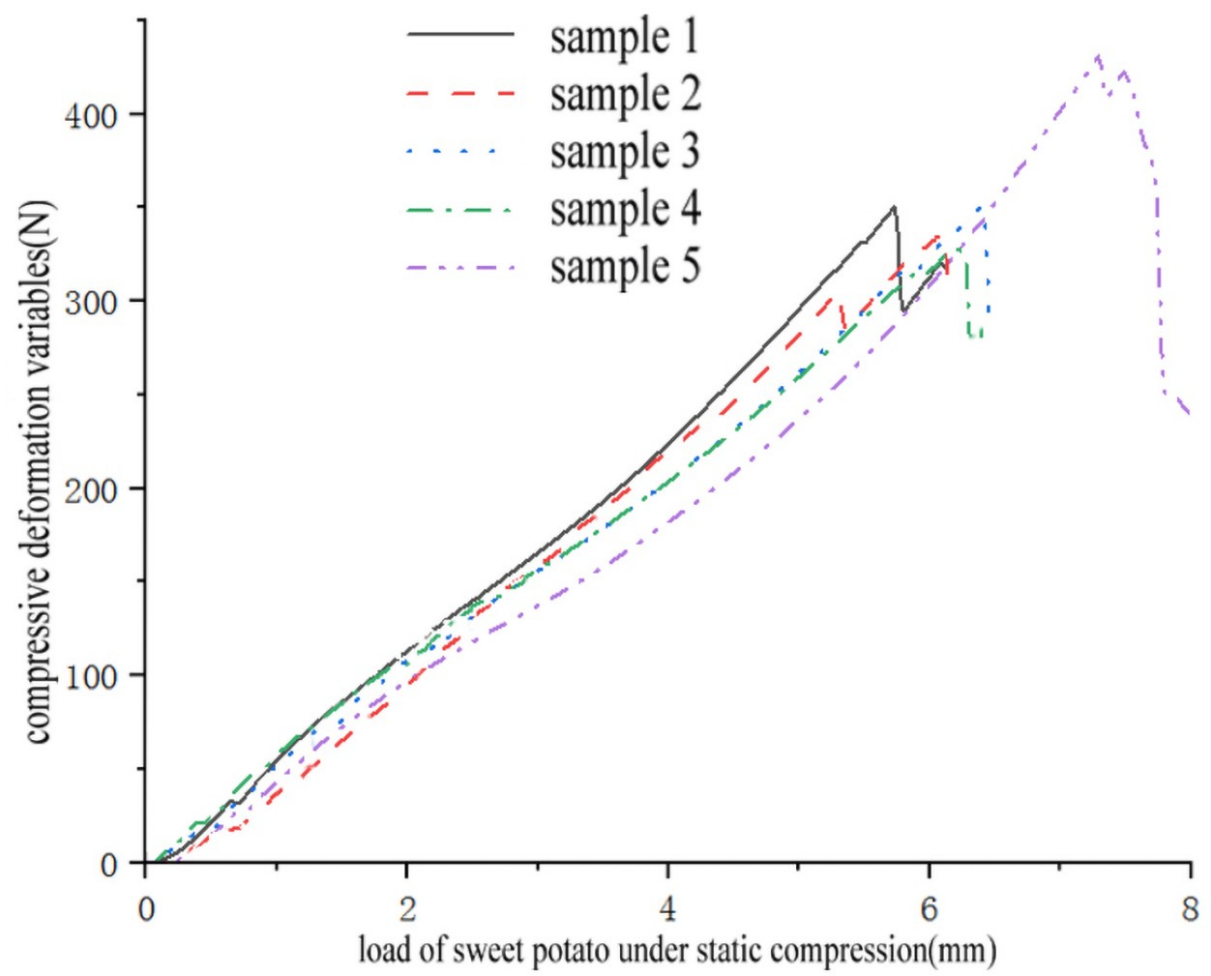
Relationship between compressive deformation variables and load of sweet potato under static compression. Data curve of sweet potato firmness test.

The pressure-displacement curve (Fig 4) was obtained from the firmness test. In the initial stage of compression, the pressure and displacement of the sweet potato block show a linear change trend, and there is no obvious yield point. Then as the load increases, the sweet potato block produces plastic deformation, and then reaches the compressive limit, the sweet potato block is crushed, and a load appears. Sudden drop phenomenon. Calculate the firmness of the five samples from formula 1, and take the average value to obtain the firmness of " sweet potato Su 16 "*σ*_*pole*_ = 2.0768MPa.

Through the drop impact test, the samples were dropped freely from different heights of 100 mm, 200 mm, 300 mm, 400 mm and 500 mm respectively with 65Mn steel, rubber plate, plastic basket, sweet potato and soil block for impact test, so that the sweet potato fell freely from the height scale position to the impact material in the long axis horizontal state. The test platform recorded the impact force *f*-time *t* data during the impact process of sweet potato. The experiment was repeated 10 times for each group, and record the average value of the area of epidermal destruction and damage. After taking the average of each group, take the group of the original data that is closest to the average value and the signal curve feedback is good for data analysis. The study analyzed the relationship between the physical movement characteristics and the damage of the sweet potato from the beginning of contact between the falling potato tubers and the collision material to the separation process. The experimental results are shown in Table 3. When only considered the impact of the drop height on the index, as the drop height increased, the maximum value of the test index had an increasing trend. When only considered the size of sweet potatoes, there was the same trend. In the following, equation fitting was performed on some test indicators and factor levels, and critical values were solved.

**Table 3:** Results of single factor experiment.

Factor	Level	Test index					
		Peak force *F*/(N)	Peak acceleration *a*/ (m·s^-2^)	Maximum deformation *s*/(mm)	Maximum average impact stress σ/(MPa)	Area of epidermal destruction *Y*_*1*_/ (mm^2^)	Area of damage *Y*_*2*_/ (mm^2^)
Drop height/mm	100	377.18	-1247.48	2.21	0.7131	0	0
	200	581.81	-1929.57	2.55	0.9705	321	0
	300	799.20	-2654.21	3.45	1.0187	513	21
	400	1054.74	-3506.00	4.12	1.0804	916	512
	500	1363.17	-4534.12	4.41	1.3101	1039	701
Collision material	65Mn steel	799.20	-2654.21	3.45	1.0187	513	21
	plastic basket	731.19	-2427.51	2.98	1.0184	437	8
	rubber plate	850.76	-2826.09	2.26	1.5463	80	0
	sweet potato	748.06	-2483.73	2.45	1.2576	212	0
	soil block	660.42	-2191.63	2.4	1.1465	256	20
Sweet potato chunk size/g	100	313.11	-3121.28	3.1	0.4769	0	0
	200	630.05	-3140.47	3.48	0.7169	253	0
	300	799.20	-2654.21	3.45	1.0187	513	21
	400	955.99	-2380.17	3.59	1.0222	868	225
	500	1513.14	-2708.67	3.74	1.0629	1145	400

### The influence of drop height on the impact characteristics of sweet potato chunks

**Fig 5:**
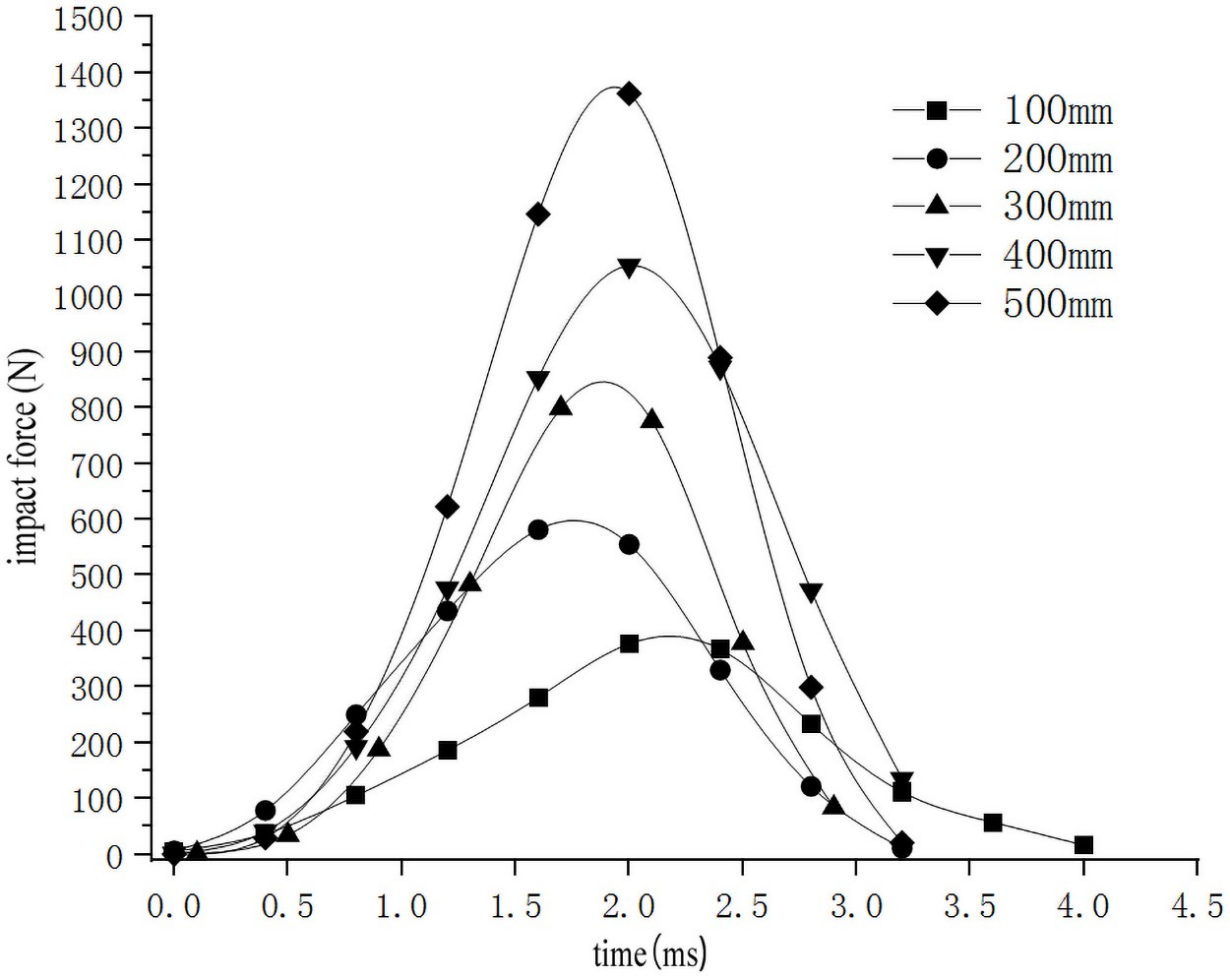
Impact force-time relationship during drop impact at different heights.

**Fig 6:**
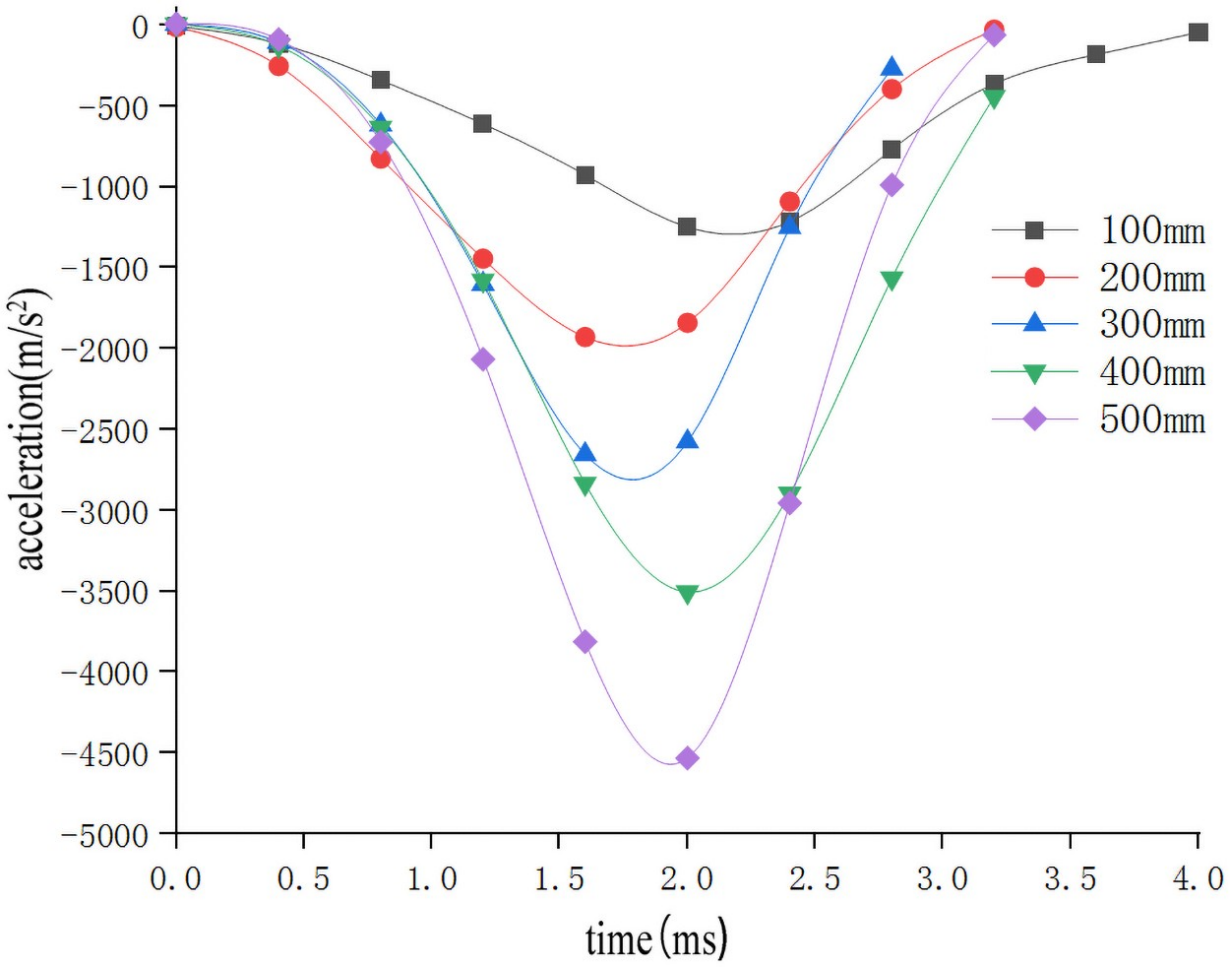
Acceleration-time relationship during drop impact at different heights.

**Fig 7:**
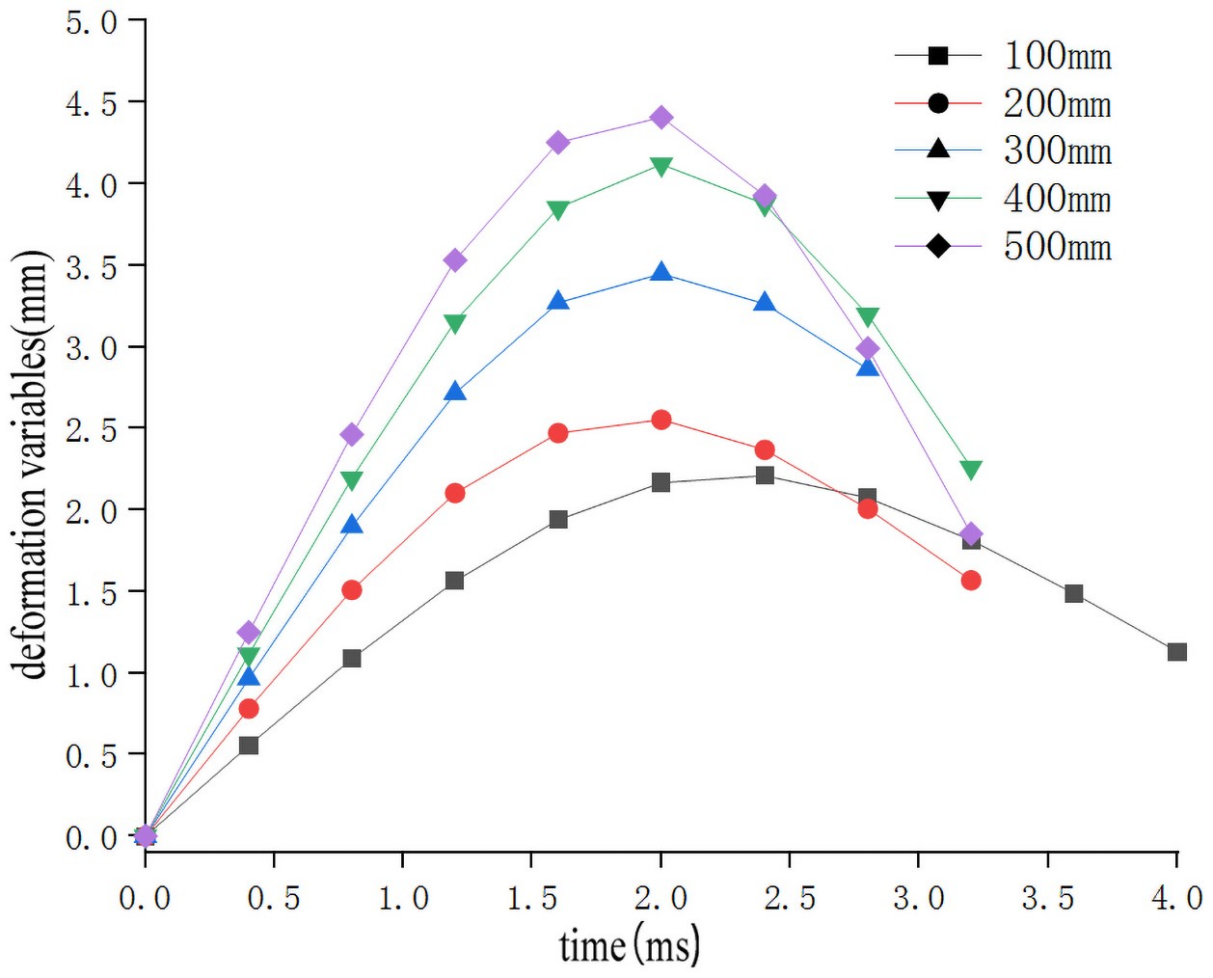
Impact deformation-time relationship during drop impact at different heights.

In order to study the influence of the drop height on the impact characteristics of the sweet potato chunks, the impact-time data on the impact of different drop heights were selected from the full factor test which the weights of the sweet potato was 300g, and the collision material was 65Mn steel(Data that is closest to the in-group mean for each group selected previously). These data constitute the single factor test analysis basis with the drop height as the independent variable. The analysis results are shown in Table 3. Use Origin software to integrate the selected test signal curve to get the impact force F-time t curve (Fig 5). According to the principle of kinematics, the acceleration a-time t curve (Fig 6) relationship diagram during the impact of falling at different heights is obtained from Eq (2). From formulas (2–6), the relationship diagram of the deformation variable s-time t curve can be obtained (Fig 7). It can be seen from Figs 5–7 that during the impact process of a free fall of the same mass of potato pieces from different heights, the peak values of impact force, acceleration, and deformation increase with the increase of the drop height. Sweet potato with a high drop height had a higher initial velocity and can reach the peak impact force faster, and because the sweet potato is an elastic plasticity body, plastic deformation absorbs part of the impact energy during the impact process, which makes the image peak appear asymmetry between left and right. Moreover, the maximum deformation produced during the drop impact process is not produced when it is subjected to the maximum impact force, and has similar stress relaxation characteristics.

**Fig 8:**
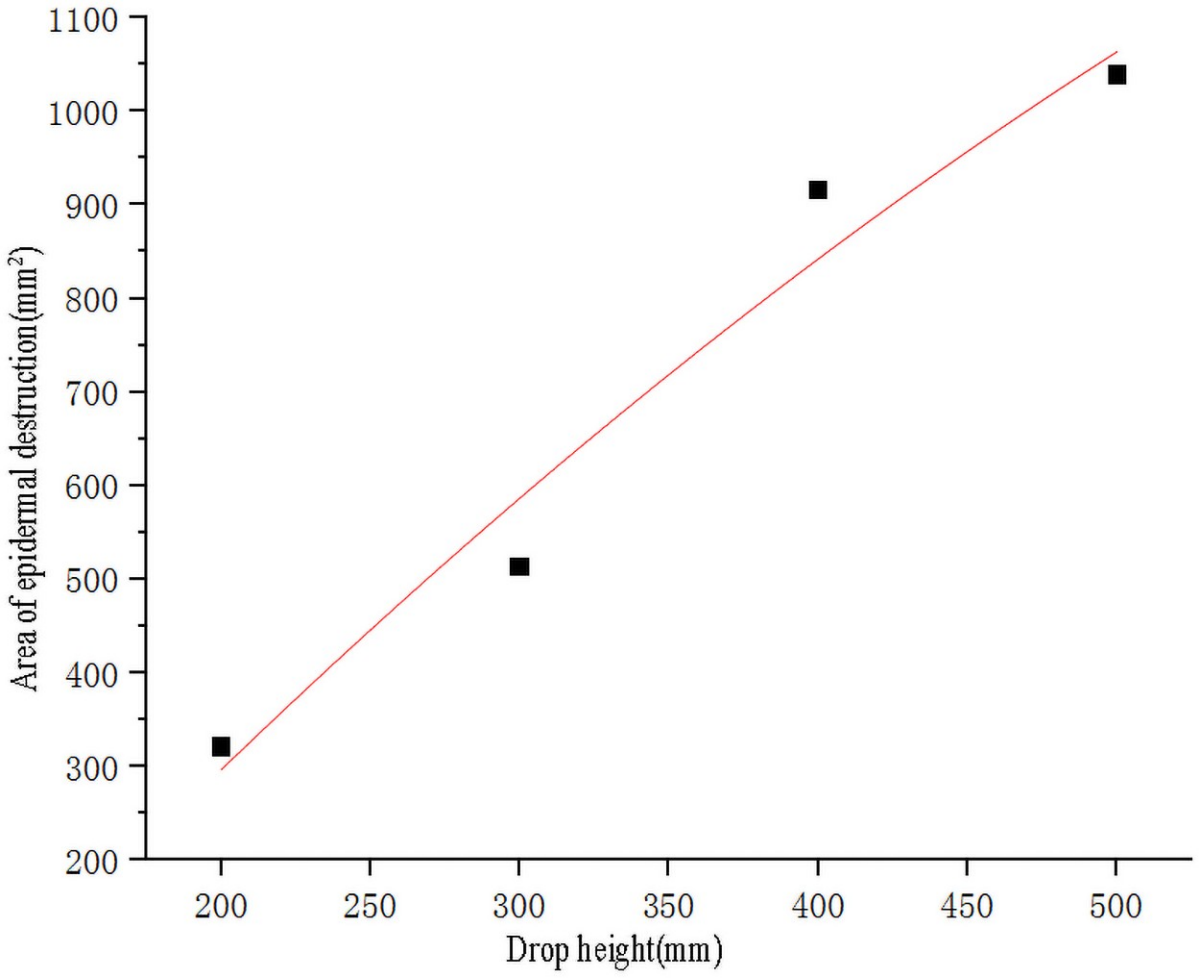
Fitting curve of sweet potato drop height and the area of epidermal destruction. Note:^1^ Goodness of Fit refers to the degree to which the regression curve fits the observed values. The statistic that measures goodness of fit is the coefficient of determination R^2^. The maximum value of R^2^ is 1. The closer R² is to 1, the better the fitting degree of the regression line to the observed value is.

Use SPSS software to fit the curve of sweet potato drop height and data (Table 3) of the area of epidermal destruction (Fig 8). The points used for the fitting are to eliminate the test points with zero epidermal destruction area. The fitting obtains the quadratic curve equation as shown in Eq (9), the goodness of fit R^2^ = 0.96^1^, and compared with we tried other forms of fitting equations, the goodness of fit of Eq 9 was closest to 1, so it had the best fitting effect. Substituting the broken skin area *Y*_*1*_ = 0 into the formula (9) to obtain the effective root, the critical epidermal destruction height of sweet potato is *h*_*1*_ = 108.28mm.


$$y_{1}=-3.1182h^{2}+2.7545h-234.2091$$ (9)

**Fig 9:**
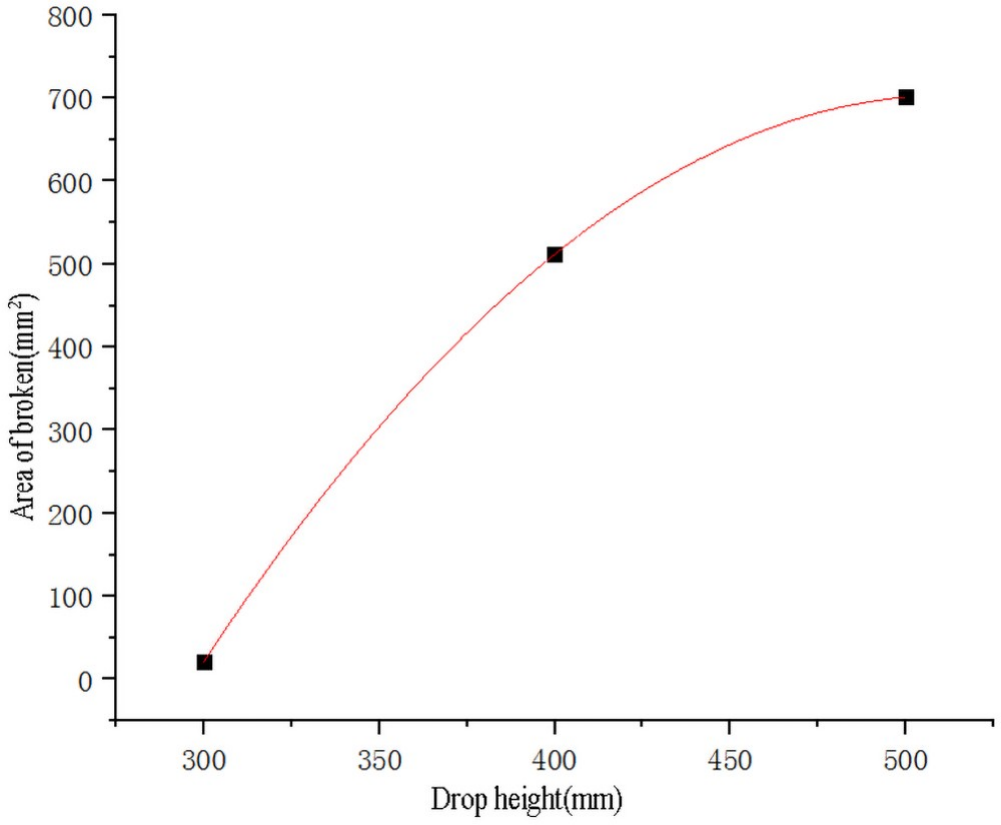
Fitting curve of sweet potato drop height and damage area.

Use SPSS software to perform curve fitting on the data (Table 3) of sweet potato drop height and the broken area (Fig 9). The points used for fitting are the test points where the broken area value is zero, and the quadratic curve Eq (10) is obtained by fitting. The goodness of fit R^2^ = 0.99, and compared with other forms of fitting equations, the goodness of fit of Eq 10 was closest to 1, so it had the best fitting effect. Substituting the broken area *Y*_*2*_ = 0 into the formula (10) to obtain the effective root, the critical damage height of sweet potato is *h*_*2*_ = 296.75mm.


$$y_{2}=-0.0151h^{2}+15.48h-3264$$ (10)

**Fig 10:**
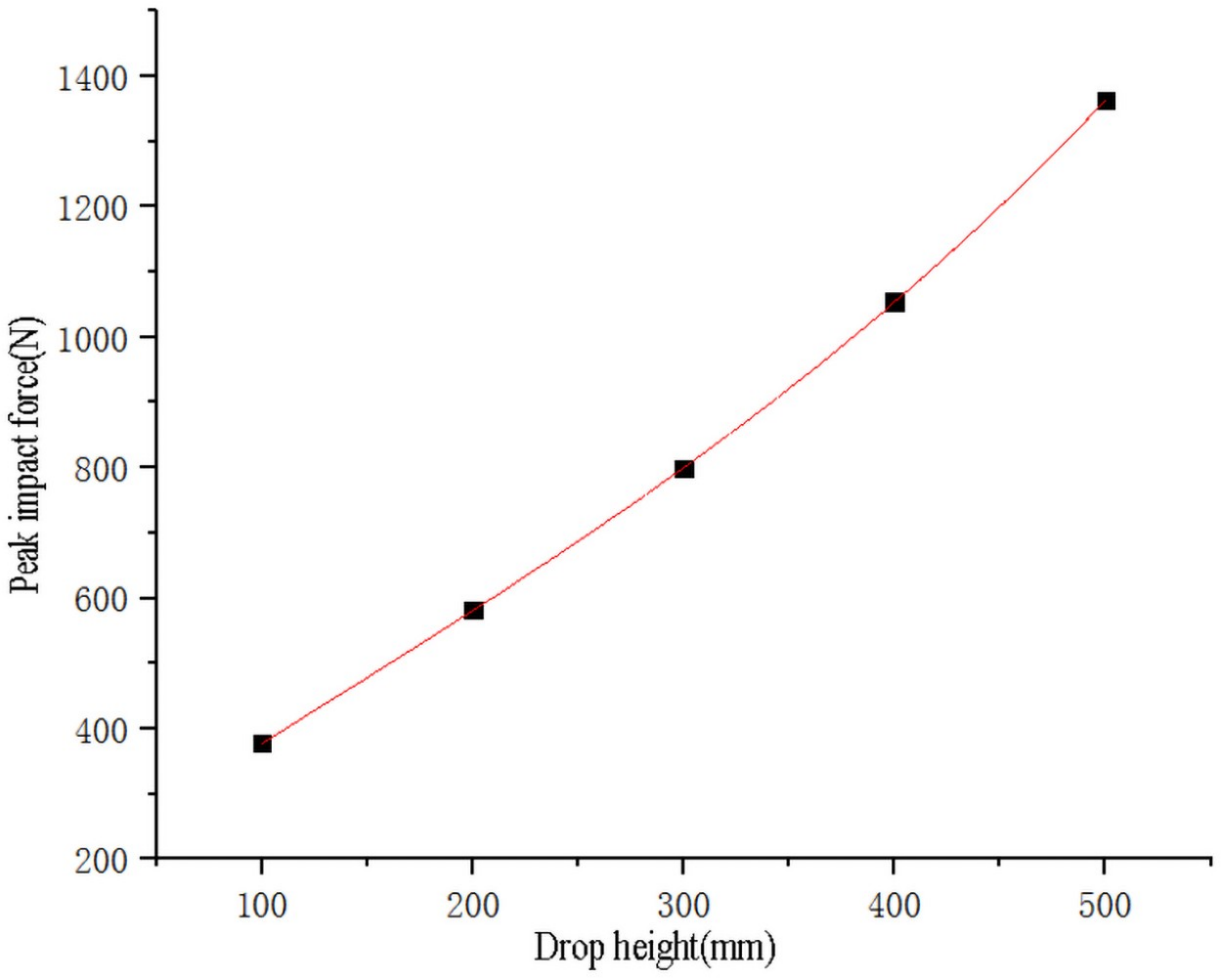
Fitting curve of sweet potato drop height and impact force peak value.

**Fig 11:**
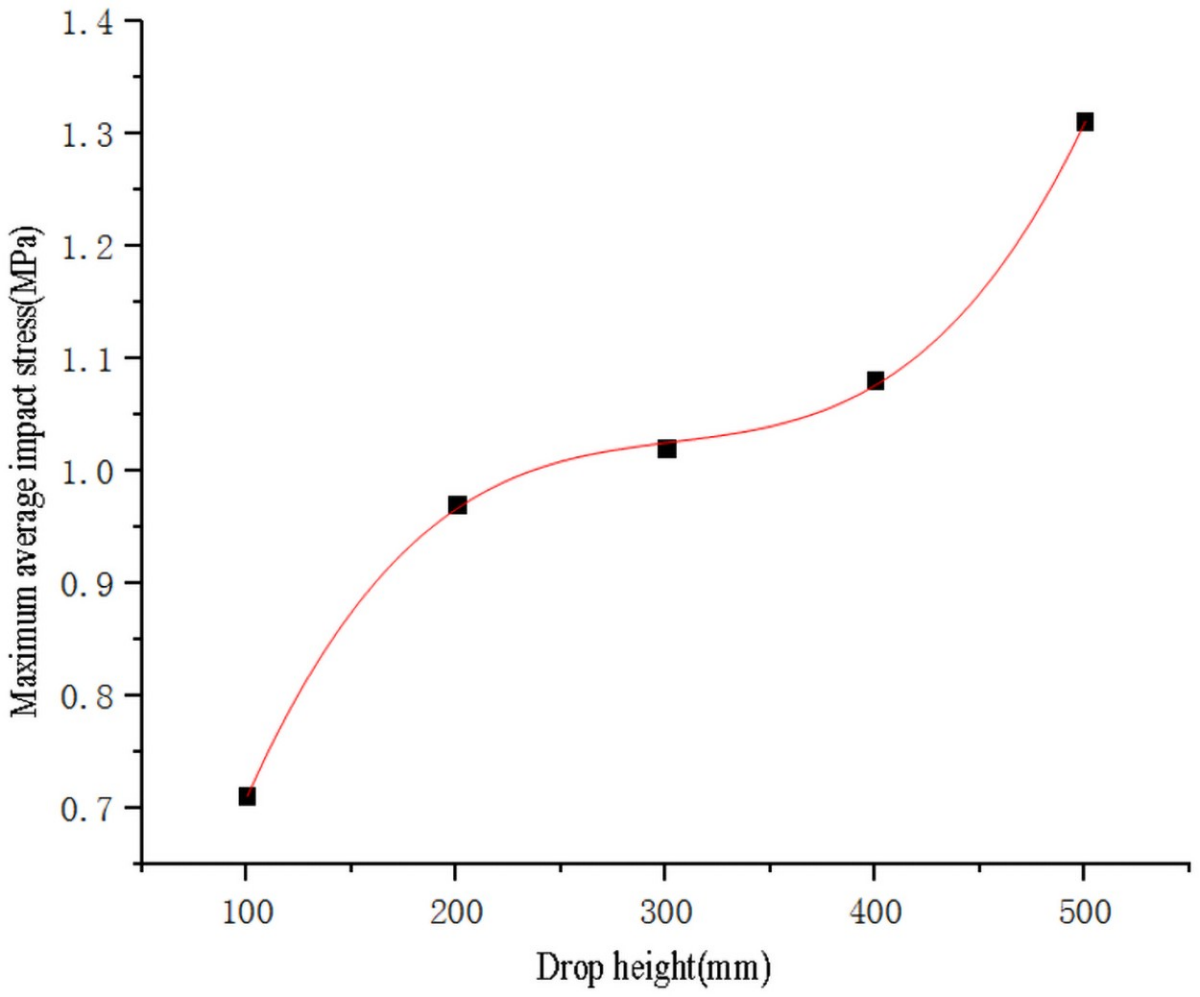
Fitting curve of sweet potato drop height and impact stress.

Use SPSS software to fit the curve of sweet potato drop height with impact force peak value (Fig 10) and maximum stress value data (Fig 11) respectively. The curve equations obtained by fitting are shown in Eqs (11) and (12), and the goodness of fit R_1_^2^ = 0.99 and R_2_^2^ = 0.99. Substitute the critical epidermal destruction height *h*_*1*_ = 108.28mm and the critical damage height *h*_*2*_ = 296.75mm into Eqs (11) and (12) respectively to obtain the critical epidermal destruction impact force value *F*_*11*_ = 395.05N, the critical epidermal destruction impact stress *σ*_*11*_ = 0.7443 MPa, the critical damage impact force *F*_*12*_ = 795.34N and the critical damage impact stress *σ*_*12*_ = 1.0251MPa, it is found that the critical damage impact stress is less than the sweet potato firmness *σ*_*pole*_ = 2.0768MPa. Therefore, it is speculated that because the surface of the sweet potato is spherical, the deformation of the first contact point of the sweet potato is the largest when it is pressed by a flat object, which will cause stress concentration, making the center contact stress greater than the edge contact stress, resulting in damage, and the resulting critical damage impact stress is the contact surface Average value of impact stress.


$$F\left(h\right)=3.3442\times 10^{-6}h^{3}-1.25\times 10^{-3}h^{2}+2.189h+168.44$$ (11)

$$\sigma \left(h\right)=3.1667\times 10^{-8}h^{3}-2.8857\times 10^{-5}h^{2}+0.009h+0.068$$ (12)

In addition, from the analysis of variance of the single factor test results of the drop height, Table 4 shows that the drop height has a very significant impact on the mechanical damage of the potato pieces (P<0.01). The safe drop height of sweet potato has important reference value for the design of related parameters such as throwing sweet potato height of separation mechanism of sweet potato excavator and falling sweet potato height of collecting sweet potato mechanism of combine harvester. Therefore, in order to reduce the damage caused by the extrusion, collision and impact of the sweet potato caused by the soil block, sweet potato and chain bar, the falling height of the separation mechanism and the parameters of the conveying separator should be comprehensively considered in the design process.

**Table 4:** Variance analysis of the influence of sweet potato falling height on various indexes.

Source		Sum of squares	Degree of freedom	Mean squares	*F* value	*P* value
area of epidermal destruction *Y*_*1*_	Within the group	7157638.400	4	1789409.600	2505.739	0.000**
	Between groups	32135.600	45	714.124	-	-
	total	7189774.000	49	-	-	-
area of damage *Y*_*2*_	Within the group	3702379.680	4	925594.920	3585.724	0.000**
	Between groups	11616.000	45	258.133	-	-
	total	3713995.680	49	1789409.600	-	-

Note: *P*<0.01 (highly significant, **); 0.01≤*P*<0.05 (significant, *).

### The influence of collision materials on the impact characteristics of potato chunks

**Fig 12:**
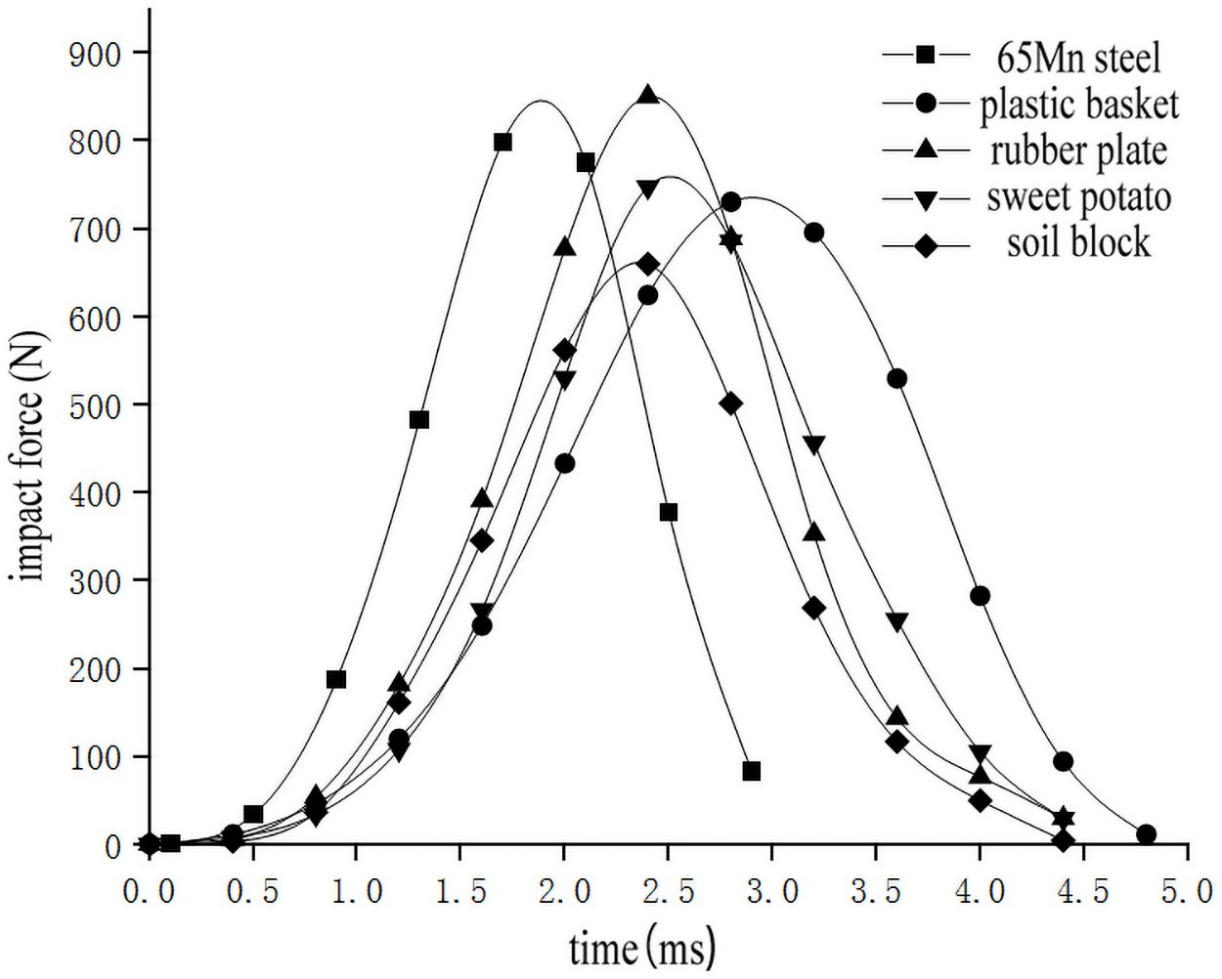
Impact force-time relationship when impacting with different collision materials.

**Fig 13:**
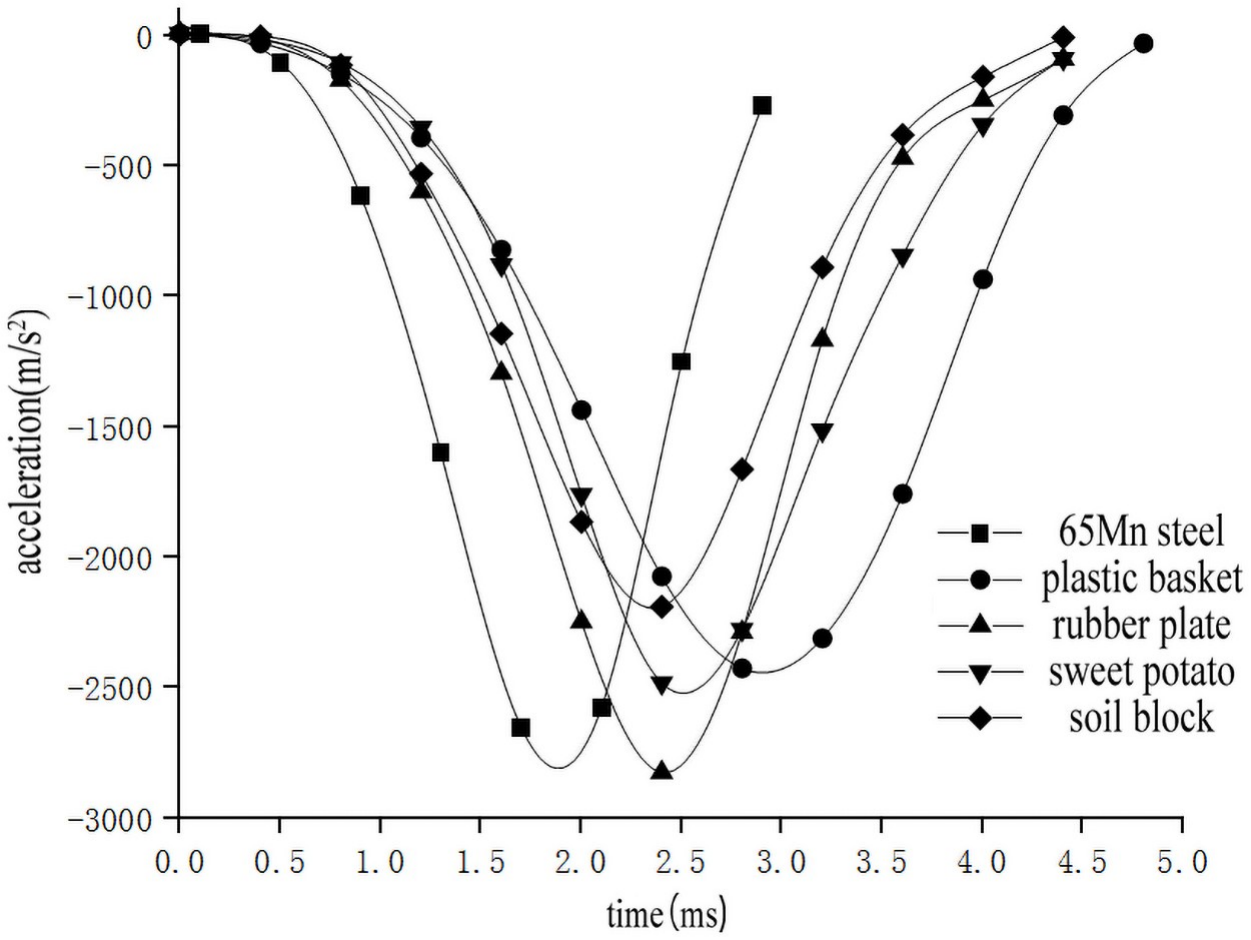
Acceleration-time relationship when impacting with different collision materials.

**Fig 14:**
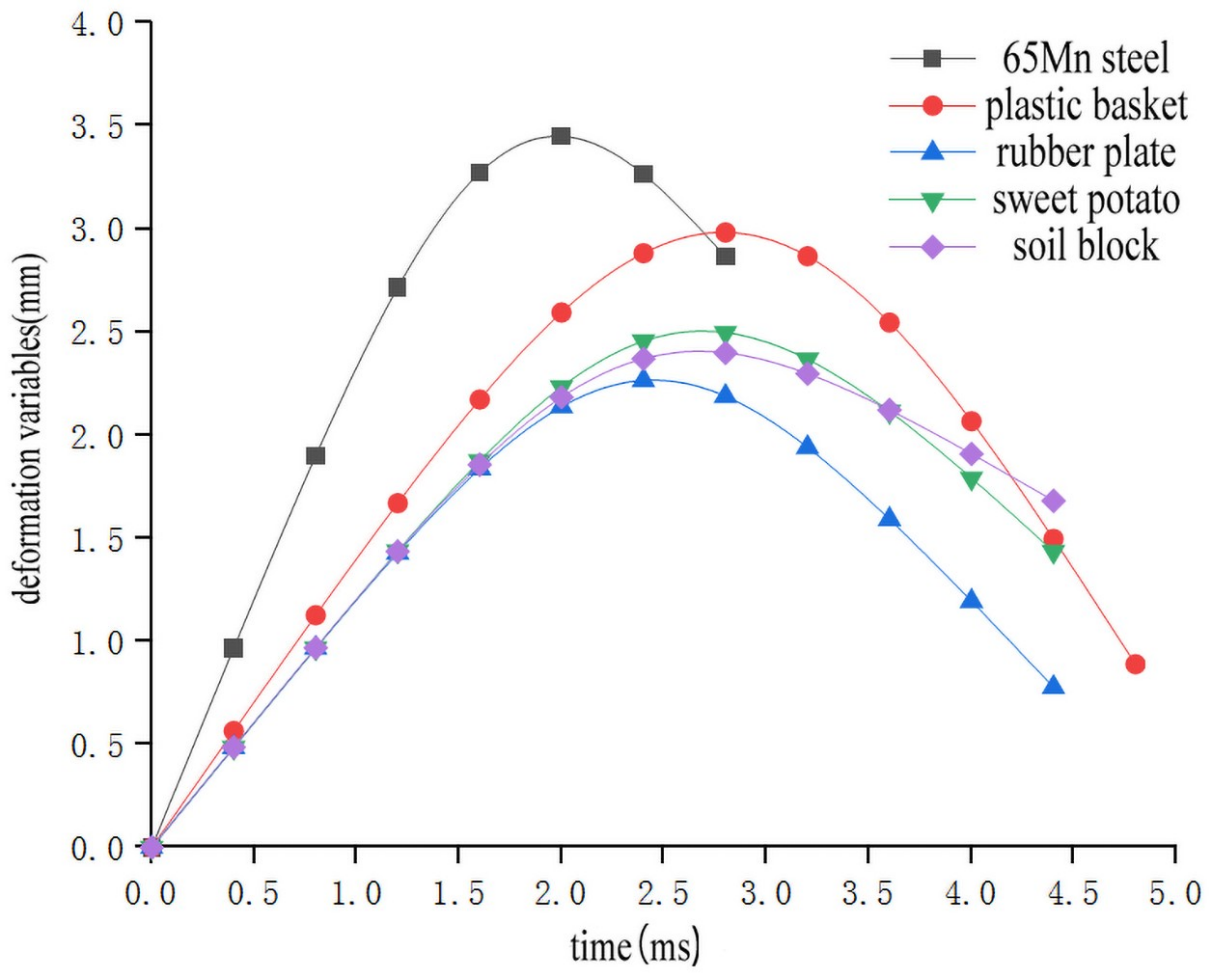
Impact deformation-time relationship when impacting with different collision materials.

In order to study the influence of collision materials on the impact characteristics of sweet potato, the impact-time data on the impact of different collision materials for the sweet potato were selected from the full factor test which the weights of the sweet potato was 300g, and the drop height was 300mm(Data that is closest to the in-group mean for each group selected previously). These data constitute the single factor test analysis basis with the impact characteristics as the independent variable. The results are shown in Table 3. Use Origin software to integrate the selected test signal curve to get the impact force *F*-time *t* curve (Fig 12). According to the kinematic formula (2), calculate and draw the acceleration *a*-time *t* curve relationship diagram during a drop impact at different heights (Fig 13). The deformation variable *s*-time *t* curve (Fig 14) relationship diagram is obtained by formulas (2–6). It can be seen from Figs 12–14 that during the impact process of a sweet potato of the same mass falling freely from the same height to different contact objects, the peak values of the impact force and acceleration are similar, but the time to reach the peak is different due to the different elasticity of the collision material. The 65Mn steel with the worst elasticity reaches the peak earliest, and the plastic basket with the strongest elasticity takes longer to reach the peak. Since the 65Mn steel material has the largest hardness, the sweet potato has the largest amount of deformation. But the hardness of soil block and rubber is small, and it will deform and absorb part of the impact energy during impact, and the contact area will increase, which will play a certain buffering effect.

According to the variance analysis of the single factor test results of the collision material (Table 5), the collision material has a significant effect on the fall damage of sweet potato (P<0.05). And through multiple comparisons between groups, it can be seen from Tables 6 and 7 that the effects on the area of sweet potato epidermal destruction are significant to each other, that is, the effects of different materials on sweet potato peeling are different, and the degree of influence is 65Mn steel>plastic basket>sweet potato>soil block >Rubber; 65Mn steel, plastic baskets and soil blocks have more significant influence on the degree of damage to sweet potatoes than rubber and sweet potatoes. The granular particles in the soil blocks make it easier to concentrate the stress during the impact of the sweet potato, thereby causing damage. Therefore, the design of the sweet potato and soil block separation mechanism should minimize the collision between the sweet potato and the 65Mn steel metal material. The appropriate amount of rubber strips and soil on the separation rod will protect the sweet potato and reduce the damage of the sweet potato. Choice will also have an impact on sweet potato damage.

**Table 5:** Variance analysis of the influence of sweet potato falling height on various indexes.

Source		Sum of squares	Degree of freedom	Mean squares	*F* value	*P* value
area of epidermal destruction *Y*_*1*_	Within the group	610424.000	4	152606.000	123.738	0.000**
	Between groups	24666.000	20	1233.300		
	total	635090.000	24			
area of damage *Y*_*2*_	Within the group	2098.400	4	524.600	3.783	0.019*
	Between groups	2773.600	20	138.680		
	total	4872.000	24			

Note: *P*<0.01 (highly significant, **); 0.01≤*P*<0.05 (significant, *).

**Table 6:** Multiple comparisons of the influence of sweet potato collision materials on the area of epidermal destruction.

material (I)	material (J)	Average difference (I-J)	*P* value	95% confidence interval	
				Lower limit	Upper limit
65Mn steel	rubber plate	434.0000	0.000**	387.6691	480.3309
	plastic basket	81.0000	0.002**	34.6691	127.3309
	sweet potato	304.0000	0.000**	257.6691	350.3309
	soil block	260.0000	0.000**	213.6691	306.3309
rubber plate	plastic basket	-353.0000	0.000**	-399.3309	-306.6691
	sweet potato	-130.0000	0.000**	-176.3309	-83.6691
	soil block	-174.0000	0.000**	-220.3309	-127.6691
plastic basket	sweet potato	223.0000	0.000**	176.6691	269.3309
	soil block	179.0000	0.000**	132.6691	225.3309
sweet potato	soil block	-44.0000	0.062	-90.3309	2.3309

Note: *P*<0.01 (highly significant, **); 0.01≤*P*<0.05 (significant, *).

**Table 7:** Multiple comparisons of the influence of sweet potato collision materials on damaged area.

material (I)	material (J)	Average difference (I-J)	*P* value	95% confidence interval	
				Lower limit	Upper limit
65Mn steel	rubber plate	20.80000	0.011*	5.2638	36.3362
	plastic basket	8.60000	0.262	-6.9362	24.1362
	sweet potato	20.80000	0.011*	5.2638	36.3362
	soil block	0.80000	0.916	-14.736	16.3362
rubber plate	plastic basket	-12.2000	0.117	-27.7362	3.3362
	sweet potato	0.0000	1.000	-15.5362	15.5362
	soil block	-20.0000	0.014*	-35.5362	-4.4638
plastic basket	sweet potato	12.20000	0.117	-3.3362	27.7362
	soil block	-7.80000	0.307	-23.3362	7.7362
sweet potato	soil block	-20.0000	0.014*	-35.5362	-4.4638

Note: *P*<0.01 (highly significant, **); 0.01≤*P*<0.05 (significant, *).

### The influence of the sweet potato chunk size on the impact characteristics

**Fig 15:**
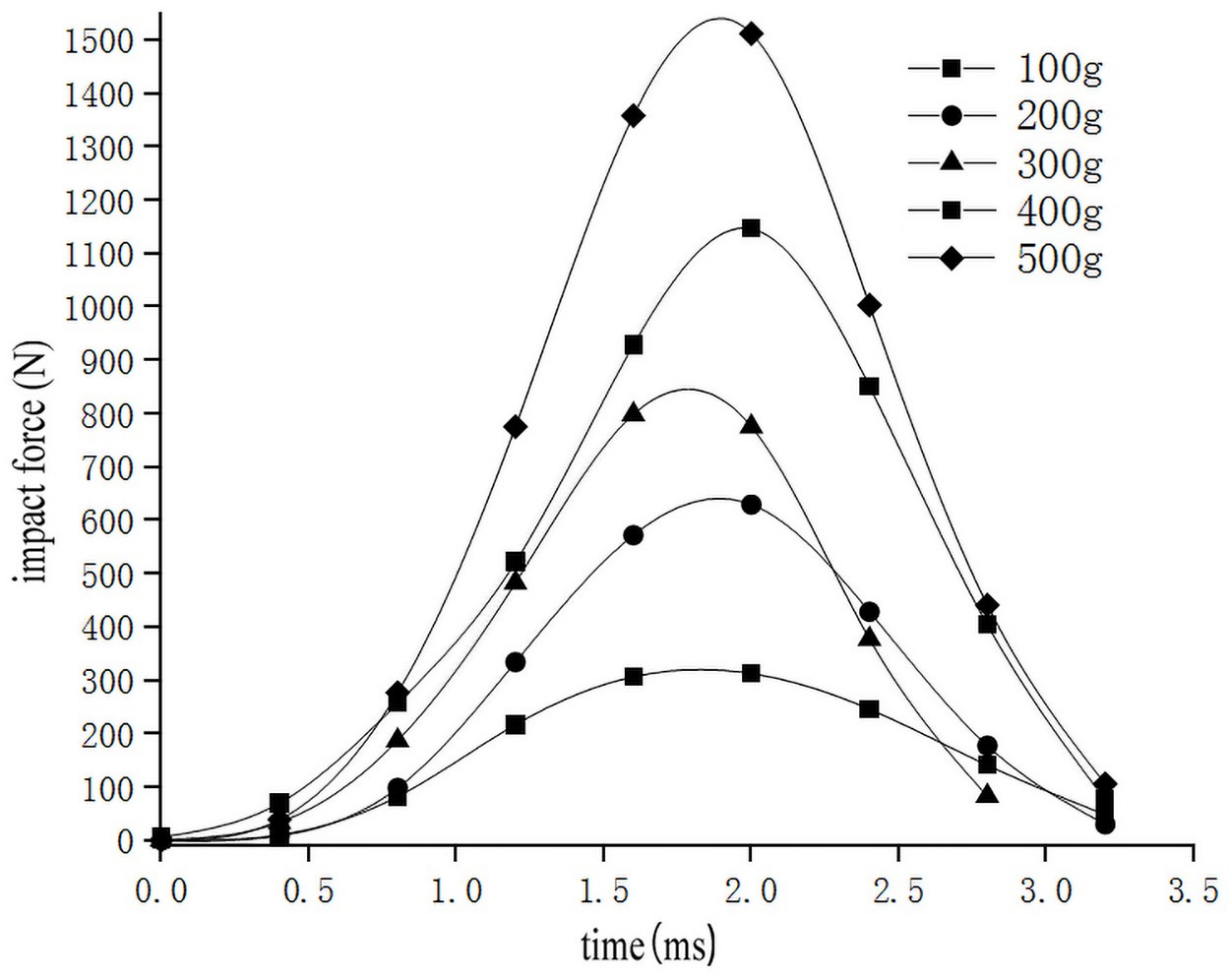
Impact force-time relationship of different sizes of sweet potato falling.

**Fig 16:**
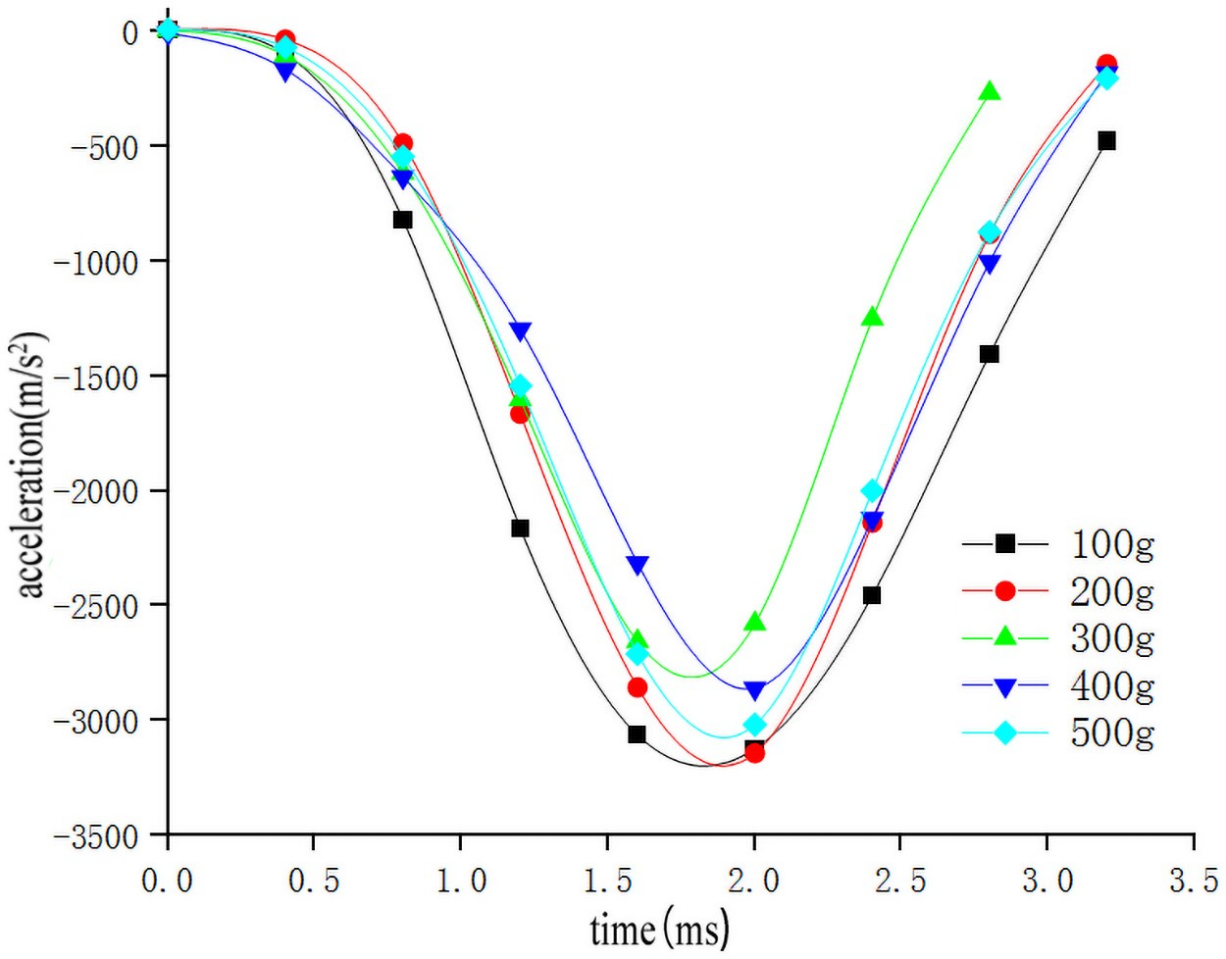
Acceleration-time relationship of different sizes of sweet potato falling.

**Fig 17:**
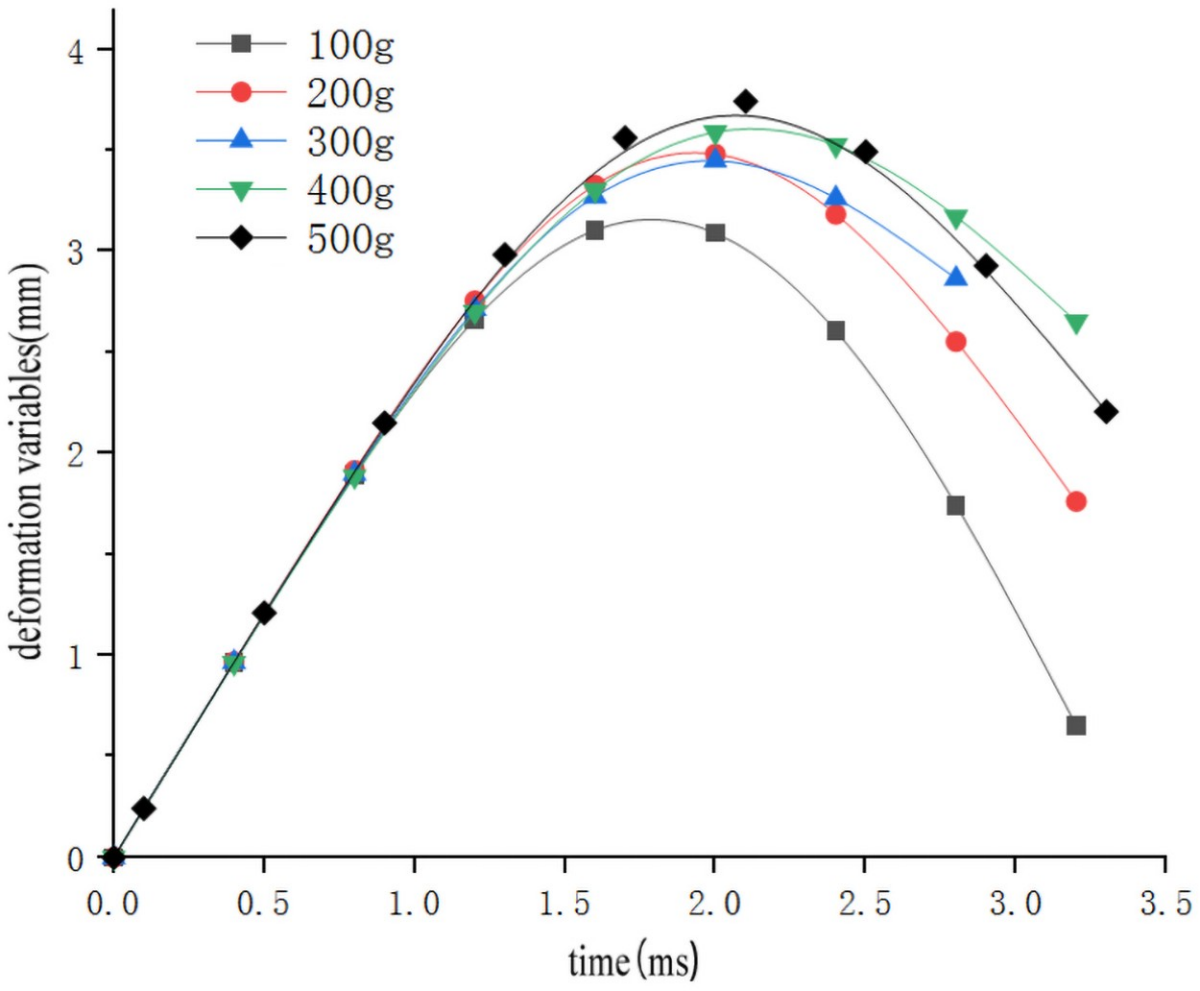
Impact deformation-time relationship of different sizes of sweet potato falling.

In order to study the influence of sweet potato chunk size on the drop impact characteristics of the sweet potato chunks, the impact-time data on the impact of different weights of the sweet potatoes were selected from the full factor test which the drop height was 300mm and the collision material was 65Mn steel(Data that is closest to the in-group mean for each group selected previously). These data constitute the single factor test analysis basis with the size of sweet potato as the independent variable. The results are as follows Table 3 shows. The original data of the selected test signals are integrated using origin to obtain the impact force *F*-time *t* curve (Fig 15). According to the kinematic formula (2), calculate and draw the acceleration *a*-time *t* curve (Fig 16) relationship diagram when falling impact at different heights. Calculate and draw the deformation variable *s*-time *t* curve (Fig 17) relationship diagram during the movement can be obtained from formulas (2–6). It can be seen from Figs 15–17, that during the impact process of free fall of different masses of sweet potato chunks from the same height, the peak values of impact force and deformation increase with the increase of the mass of sweet potato chunks. However, the peak value of acceleration has no obvious correlation with the comprehensive effect of impact force and mass, and its peak value has little difference.

**Fig 18:**
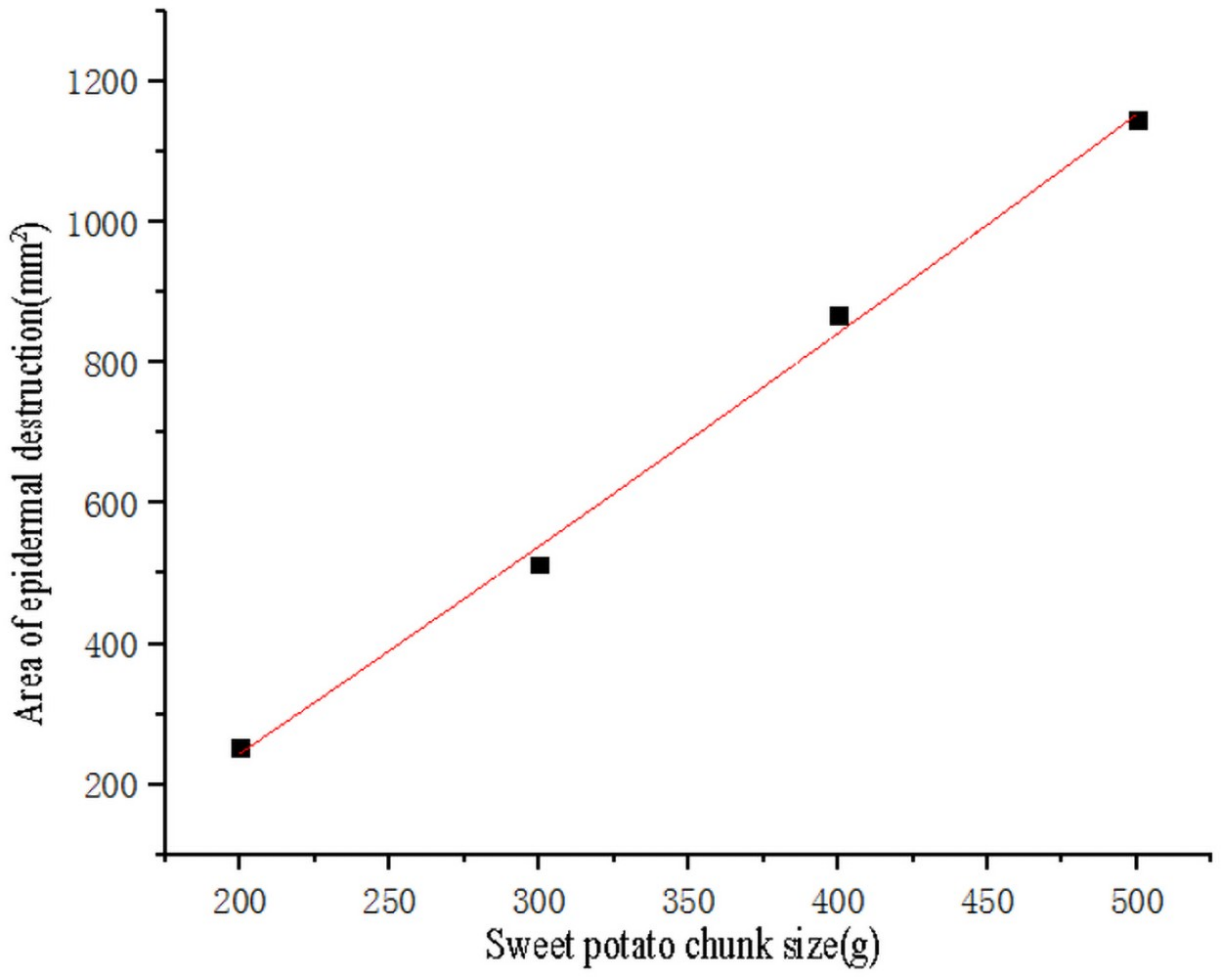
Fitting curve of sweet potato chunk size and the area of epidermal destruction.

Use SPSS software to perform curve fitting on the quality of sweet potato chunks and data (Table 3) of the area of epidermal destruction (Fig 18), the points used for the fitting are to eliminate the test points with zero area of epidermal destruction. The fitting obtains the quadratic curve equation (Eq 13), the goodness of fit R^2^ = 0.99, and the fitting effect was better than any other form of fitting equations. Substituting the area of epidermal destruction *Y*_*1*_ = 0 into the formula (13) to obtain the effective root, the critical epidermal destruction mass of sweet potato *m*_*1*_ = 114.78g.


$$y=4.25\times 10^{-4}m^{2}+2.7335m-319.35$$ (13)

**Fig 19:**
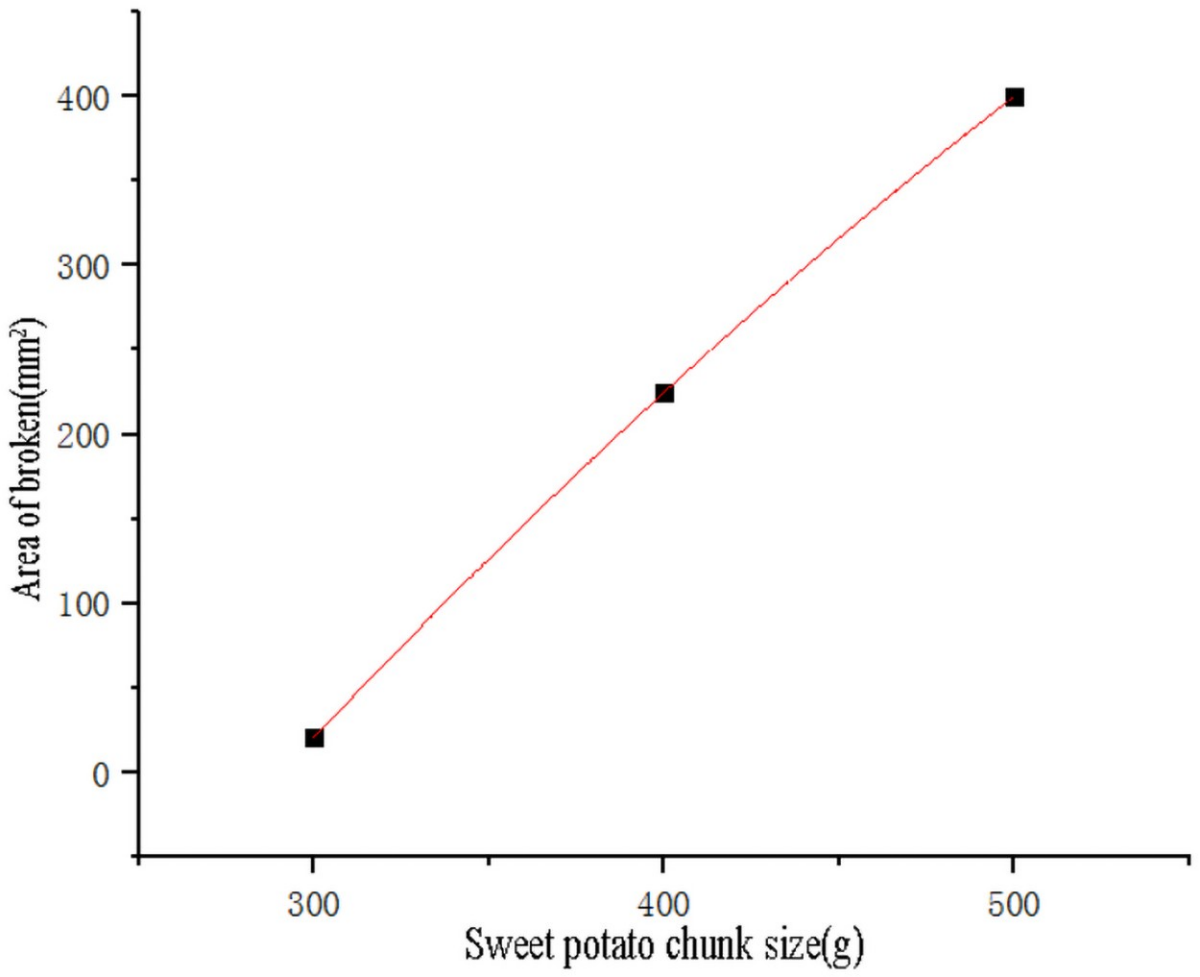
Fitting curve of sweet potato chunk size and the area of damage.

Use SPSS software to perform curve (Fig 19) fitting on the quality of sweet potato chunks and broken area data (Table 3). The points used for the fitting are to eliminate the test points with zero broken area. The fitting obtains the quadratic curve Eq (14)), the goodness of fit R^2^ = 0.99, and the fitting effect was best. Substituting the broken area *Y*_*2*_ = 0 into formula (14) to obtain the effective root, the critical damage mass of sweet potato *m*_*2*_ = 290.45g.


$$y=-1.45\times 10^{-3}m^{2}+3.055m-765$$ (14)

**Fig 20:**
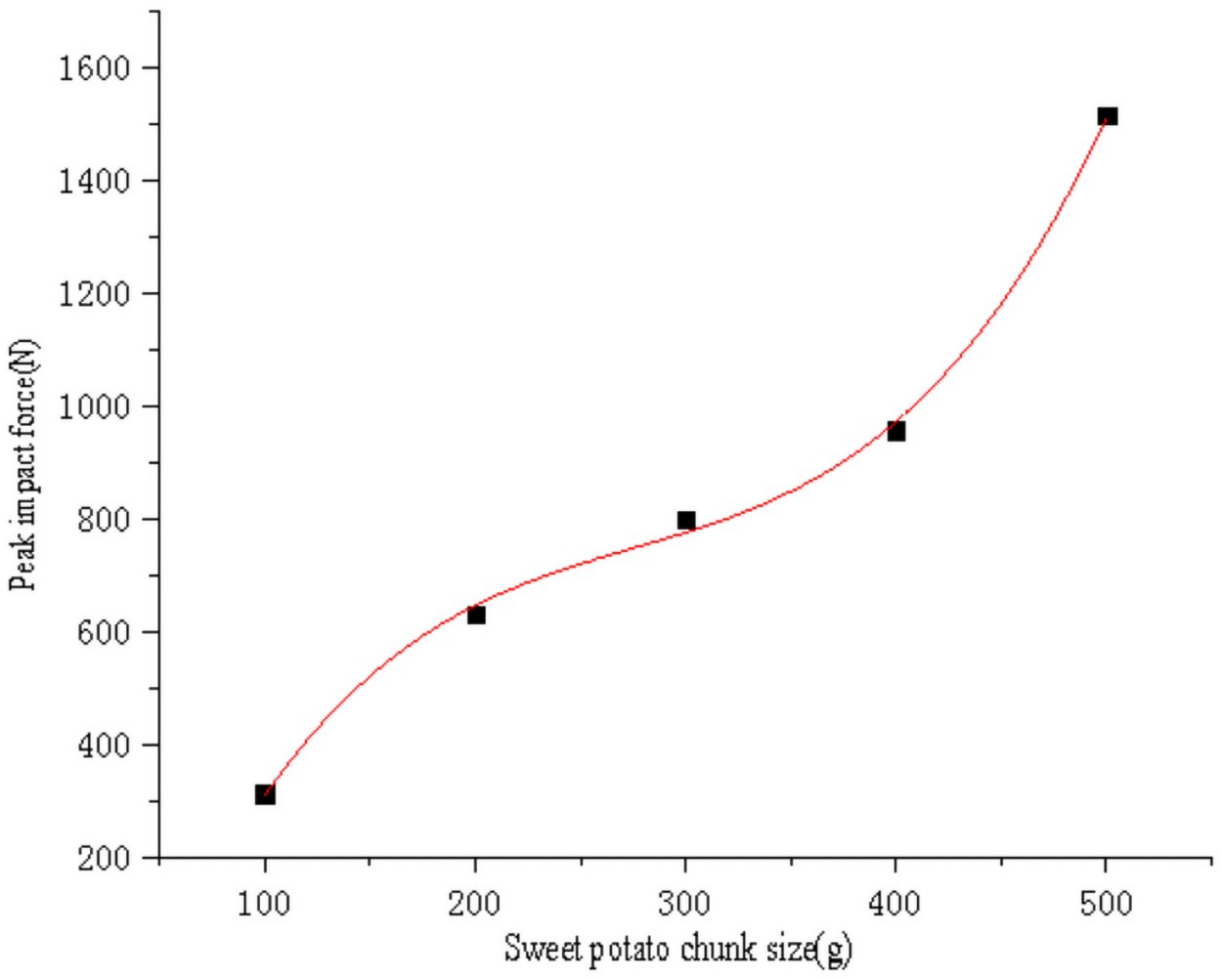
Fitting curve of sweet potato chunk size and impact force peak value.

**Fig 21:**
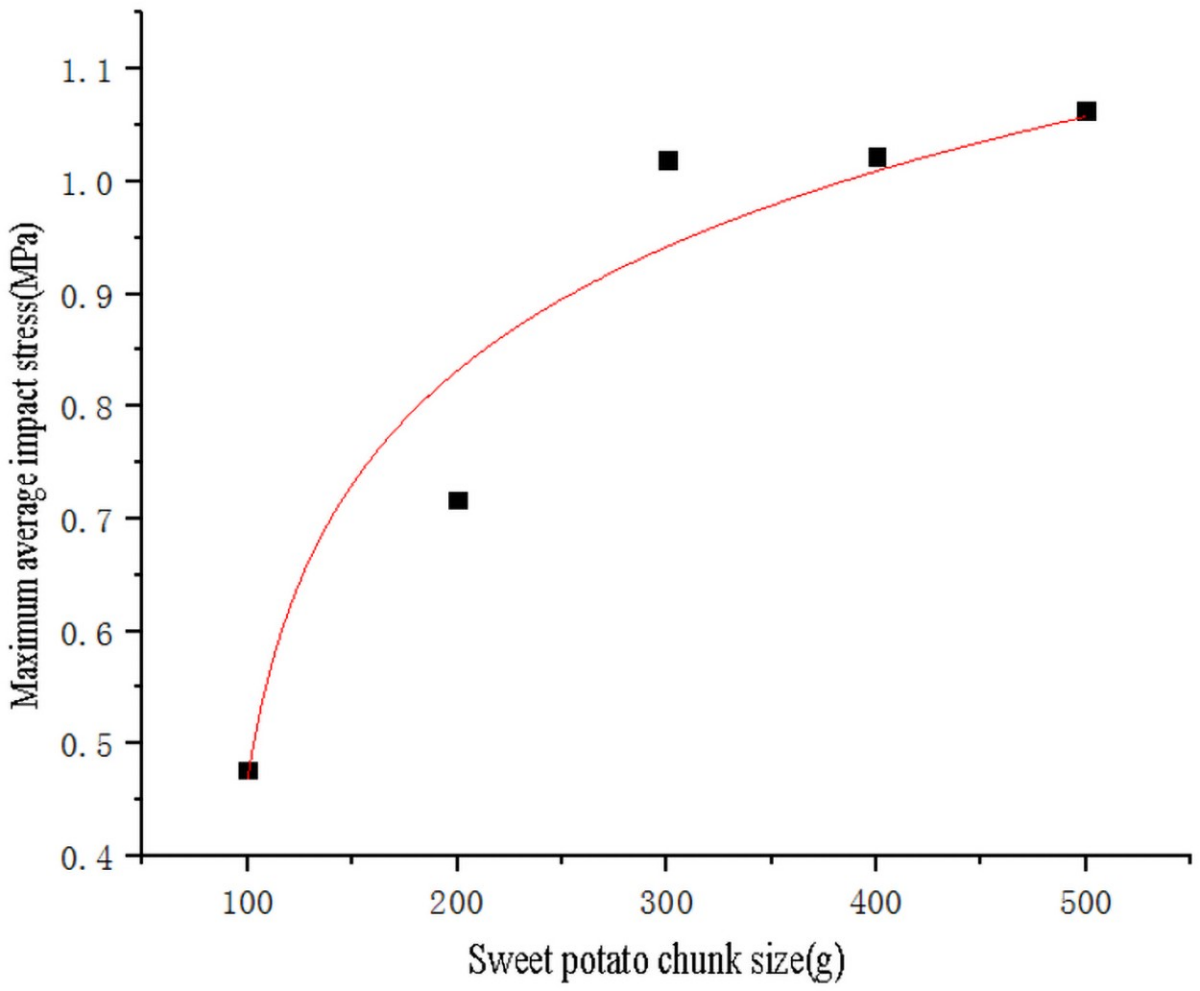
Fitting curve of sweet potato chunk size and impact stress.

Use SPSS software to fit the curve of sweet potato chunk size with impact force peak value (Fig 20) and maximum stress value data (Fig 21) respectively. The curve equations obtained by fitting are shown in Eqs (15) and (16), and the goodness of fit R_1_^2^ = 0.99 and R_2_^2^ = 0.99. Substituting the critical epidermal destruction mass *m*_*1*_ = 114.78g and critical damage mass *m*_*2*_ = 290.45g into Eqs (15) and (16) respectively to obtain the critical epidermal destruction impact force value *F*_*21*_ = 379.52N and the critical epidermal destruction impact stress *σ*_*21*_ = 0.6394 MPa, the critical damage impact force *F*_*22*_ = 744.61N, the critical damage impact stress *σ*_*22*_ = 0.9677MPa. It is found that the obtained critical epidermal destruction impact force, critical epidermal destruction impact stress, critical damage impact force, critical damage impact stress are not much different from the values obtained by the single factor test fitting equation of sweet potato drop height. Therefore, the data are mutually verified, and the critical damage impact stress It is also less than sweet potato firmness *σ*_*pole*_ = 2.0768MPa.


$$F(m)=4.567\times 10^{-5}m^{3}-0.038m^{2}+11.5m-508.882$$ (15)

$$\sigma \left(m\right)=0.181ln\left(m-80.566\right)$$ (16)

The results of the drop characteristics test of the size of the sweet potato chunks show that when the quality of the sweet potato chunks increases, the peak impact force, the peak acceleration and the maximum deformation increase. The higher the quality of the sweet potato chunks, the greater the amount of deformation when it falls and collides with the collision material, and the energy absorbed by the sweet potato chunks during the impact will also increase, resulting in increased damage to the sweet potato chunks. Through the analysis of variance of the single factor test results of sweet potato chunk quality, Table 8 shows that the quality of sweet potato has a significant impact on the drop impact characteristics of potato chunks (P<0.01). Therefore, during the mechanical harvest of sweet potato, the quality distribution of sweet potato is estimated in advance according to the variety of sweet potato, and then the parameters of the harvester are adjusted, which is of great significance to reduce the mechanical damage of sweet potato.

**Table 8:** Variance analysis of the influence of sweet potato chunk size on each index.

Source		Sum of squares	Degree of freedom	Mean squares	*F* value	*P* value
area of epidermal destruction *Y*_*1*_	Within the group	4192027.040	4	1048006.760	1057.460	0.000**
	Between groups	19821.200	20	991.060		
	total	4211848.240	24			
area of damage *Y*_*2*_	Within the group	603067.840	4	150766.960	296.692	0.000**
	Between groups	10163.200	20	508.160		
	total	613231.040	24			

Note: *P*<0.01 (highly significant, **); 0.01≤*P*<0.05 (significant, *).

## Conclusions

Aimed at the problem that sweet potato was prone to damage during mechanized harvesting, storage and transportation, this study studied the safe drop height and damage mechanism of sweet potato under different circumstances. Through the sweet potato mechanical properties test and drop impact test, the mathematical model between the drop height and impact force, impact stress, broken skin area, damaged area was established respectively. The mathematical models between the weight of sweet potato and impact force, impact stress, broken skin area and damaged area were established respectively. The relationship between the impact characteristics and the damage of the sweet potato was analyzed by calculating and investigating the change law of the velocity, acceleration, deformation, impact force and impact stress in every millisecond of the sweet potato collision process with a series of mathematical models. The experimental results showed that the size and the drop height of sweet potato tuber both had very significant effects on the test indexes. The collision material had significant influence on the mechanical damage of sweet potato collision, and the impact degree on the area of epidermal destruction was 65Mn steel > plastic basket > sweet potato > soil block > rubber. The impact of 65Mn steel, plastic basket and soil block on the damage degree of sweet potato was more significant than that of rubber and sweet potato. The granulated particles in soil block made the stress concentration in the collision process of sweet potato more easily, so as to produce damage.

Through the sweet potato firmness test, the firmness of "sweet potato Su 16" σ_*pole*_ = 2.0768MPa. The critical damage impact stress of sweet potato was less than the firmness, indicated the existence of stress concentration phenomenon. Therefore, the critical damage stress measured was the average impact stress of the contact surface. Through the drop impact test of sweet potato, the experimental data were analyzed theoretically, and obtained the curve of time with impact force, acceleration, and impact deformation respectively when the sweet potato dropped at different heights/with different quality, and a mathematical model was established. Finally, when 300g sweet potato collided with 65Mn steel, the critical epidermal destruction impact force value of sweet potato F_11_ = 395.05N, the critical epidermal destruction impact stress σ_11_ = 0.7443 MPa, the critical damage impact force F_12_ = 795.34N, the critical damage impact stress σ_12_ = 1.0251MPa. And when the sweet potato fell at a height of 300mm and collides with 65Mn steel, the critical epidermal destruction impact force value of sweet potato was F_21_ = 379.52N, the critical epidermal destruction impact stress σ_21_ = 0.6394 MPa, the critical damage impact force F_22_ = 744.61N, the critical damage impact stress σ_22_ = 0.9677MPa. Compared with acceleration signal acquisition by Xie Shengshi [19] and shape variable signal analysis by Hong Xiang [23], impact signal acquisition is more accurate and reliable.

In the test, at the moment of collision, the center of gravity may not been vertically above the landing point, which caused the sweet potato block to rotate, and transforms part of the impact energy into rotational kinetic energy. The drop impact platform built in the experiment can measured the energy absorbed by the potato after reducing. Because the impact sensor was mounted only in the vertical direction, it only measures the vertical impact force and did not take into account the scratches along the tangential direction of the sweet potato cuticle. Since the collision of sweet potato tuber in the actual production process is not always along the vertical direction, it is necessary to consider the relationship between the force on the tangent direction of sweet potato tuber and the scratch in the subsequent research.

The research methods and conclusions of this paper are of great significance for us to understand the damage mechanism of sweet potato in the harvest process and provide data reference and theoretical basis for the design and optimization of sweet potato harvesting machinery, which will effectively reduce the damage problem of sweet potato in the mechanized harvest process and promote the mechanization of sweet potato industry in the world.

## Supporting information

S1 Table Physical properties of collision materials. Material parameters can be measured by special testing instruments or obtained by referring to material parameter manual.
(XLSX)

S2 Table Levels of test factors. The table lists five levels of parameter setting for the three factors in the test.
(XLSX)

S3 Table Results of single factor experiment. (XLSX)

S4 Table Variance analysis of the influence of sweet potato falling height on various indexes. Note: P<0.01 (highly significant, **); 0.01≤P<0.05 (significant, *).
(XLSX)

S5 Table Variance analysis of the influence of sweet potato falling height on various indexes. Note: P<0.01 (highly significant, **); 0.01≤P<0.05 (significant, *).
(XLSX)

S6 Table Multiple comparisons of the influence of sweet potato collision materials on the area of epidermal destruction. Note: P<0.01 (highly significant, **); 0.01≤P<0.05 (significant, *).
(XLSX)

S7 Table Multiple comparisons of the influence of sweet potato collision materials on damaged area. Note: P<0.01 (highly significant, **); 0.01≤P<0.05 (significant, *).
(XLSX)

S8 Table Variance analysis of the influence of sweet potato chunk size on each index. Note: P<0.01 (highly significant, **); 0.01≤P<0.05 (significant, *).
(XLSX)
